# Modeling the effects of EMT-immune dynamics on carcinoma disease progression

**DOI:** 10.1038/s42003-021-02499-y

**Published:** 2021-08-18

**Authors:** Daniel R. Bergman, Matthew K. Karikomi, Min Yu, Qing Nie, Adam L. MacLean

**Affiliations:** 1 Department of Mathematics, University of California, Irvine, CA USA; 2 USC Norris Comprehensive Cancer Center, Keck School of Medicine of the University of Southern California, Los Angeles, CA, USA; 3 Department of Stem Cell Biology and Regenerative Medicine, Keck School of Medicine, University of Southern California, Los Angeles, CA USA; 4 Department of Cell and Developmental Biology, University of California, Irvine, CA USA; 5 Department of Quantitative and Computational Biology, University of Southern California, Los Angeles, CA, USA

**Keywords:** Differential equations, Cellular signalling networks, Computational models, Gene regulatory networks, Bayesian inference

## Abstract

During progression from carcinoma in situ to an invasive tumor, the immune system is engaged in complex sets of interactions with various tumor cells. Tumor cell plasticity alters disease trajectories via epithelial-to-mesenchymal transition (EMT). Several of the same pathways that regulate EMT are involved in tumor-immune interactions, yet little is known about the mechanisms and consequences of crosstalk between these regulatory processes. Here we introduce a multiscale evolutionary model to describe tumor-immune-EMT interactions and their impact on epithelial cancer progression from in situ to invasive disease. Through simulation of patient cohorts in silico, the model predicts that a controllable region maximizes invasion-free survival. This controllable region depends on properties of the mesenchymal tumor cell phenotype: its growth rate and its immune-evasiveness. In light of the model predictions, we analyze EMT-inflammation-associated data from The Cancer Genome Atlas, and find that association with EMT worsens invasion-free survival probabilities. This result supports the predictions of the model, and leads to the identification of genes that influence outcomes in bladder and uterine cancer, including FGF pathway members. These results suggest new means to delay disease progression, and demonstrate the importance of studying cancer-immune interactions in light of EMT.

## Abstract (web summary)

Bergman, Karikomi et al. propose a multiscale evolutionary model to describe tumor-immune-EMT interactions and their impact on epithelial cancer progression, which highlights a controllable region that maximizes invasion-free survival. Using EMT-inflammation associated TCGA data, the authors find EMT association worsens invasion-free survival and identify genes that influence outcomes in bladder and uterine cancer.

## Introduction

The majority of deaths from cancer are due to metastasis of the disease^1^. It is thus of critical importance to understand better the progression from in situ to invasive disease. Underlying this progression are genetic and epigenetic events, including mutations in pathways critical to the success of the cancer cell (driver mutations)^2^. These pathways include cell proliferation, apoptosis, and immunogenicity.

Cancer and the immune system interact in myriad ways. The immune system modulates the tumor microenvironment (TME), as immune signals that affect the tumor can be amplified or repressed through feedback in response to local inflammatory signals. This complex cell signaling occurs alongside the targeting (and potential eradication) of the tumor by immune cells^3^.

The effects of the immune system on a tumor can be broadly summarized into two branches. The cytotoxic branch of the immune system, such as natural killer cells (NKs) and cytotoxic T cells (CTLs), seek out and lyse tumor cells. These cells can lose efficacy or deactivate upon carrying out their effector functions or via PD1-PDL1 signaling^4,5^. The regulatory branch of the immune system (Tregs, and other factors), inhibits the effective functioning of the cytotoxic branch^6^. Inflammation can increase the probability of cancer incidence and progression, with some of the most pronounced effects seen for tumors originating in gastrointestinal and pancreatic tissues^7,8^. Recent work has shown, contrary to the typical effects of inflammation on cancer, that under certain conditions inflammation may not be oncogenic but rather oncoprotective^9^.

Immunotherapies are beginning to realize their potential, and show large impacts on patient health and survival^10,11^, and may even provide a cure for certain hematopoietic cancers via anti-CD19 CAR-T cells^12^. The presentation of antigens on tumor cells is recognized by innate immune cells that are transported to lymph nodes where T cells (and other components) can be activated^13^. The tumor also engages in processes that can indirectly modify the TME, for example by releasing transforming growth factor-beta (TGF-β), which can shift the TME towards a tumor-supportive environment by enhancing immunosuppression via activation of Tregs^13^.

Epithelial-to-mesenchymal transition (EMT) describes a reversible process by which cells displaying an epithelial phenotype transition into cells with a mesenchymal phenotype. Epithelial cells are—in part—defined by tight cell–cell adhesion. Mesenchymal cells exhibit less adhesion, greater ranges of motility, and may possess stem-like properties^14^, although controversy regarding “stemness” and EMT remains^15,16^. Recent work has shown that—rather than being a binary process—at least two stable intermediate EMT states exist^17,18^. Ongoing investigations into the plasticity and stability of EMT overlap with discussions elsewhere, e.g., of discrete vs continuous processes during cell differentiation^19^. Intermediate states have emerged as a central mechanism by which cell fates (and the noise inherent within them) can be controlled^20–22^.

Two features of the mesenchymal phenotype are of particular relevance in the context of cancer–immune interactions. (i) mesenchymal tumor cells (MTC) proliferate less than epithelial cells, we refer to this as mesenchymal growth arrest (MGA), and can be considered related to (in the sense of quiescence) the “stemness” phenotype of the MTCs^23^. (ii) mesenchymal cells are less susceptible to immune clearance^24^. As a cell is targeted by cytotoxic immune cells for clearance, a physical connection between the two cells must be established. This immunological synapse—mediated in part by T-cell receptors bound to antigens and the major histocompatibility complex on the target cell—is downregulated in mesenchymal cells, thus inhibiting the formation of the synapse^24^. We refer to this phenotype as mesenchymal immune evasion (MIE).

In addition to the prominent role it has in metastasis, EMT has more recently been shown to also regulate other aspects of tumor progression^14,25^ and tumor dormancy^26^. TGF-β, a master regulator of EMT^27^, is at once implicated heavily in tumor-mediated immune responses since Tregs release TGF-β upon arriving at the tumor site^24^. In hepatocellular carcinoma, for example, there is direct evidence linking Treg-secreted TGF-β with EMT^28^. Thus, even by considering only the TGF-β pathway, we find compelling evidence that these three core components (the tumor, the immune system, and EMT) interact. It, therefore, strikes us as a priority to develop models to understand how the interactions between each of these three components affect cancer incidence and progression. Although EMT can be induced by a host of signaling factors in addition to TGF-β, to constrain model complexity we consider only the effects of one signaling pathway in the model developed.

Mathematical oncology, that is, mathematical models of cancer incidence, progression, and treatment, has become a well-developed field; many models have offered insight into the cellular interactions underlying cancer and its interplay with the immune system, including older^29–31^ and more recent works^32–46^. These studies have increased our understanding of how tumors grow in the presence of various immune components, and how treatment regimes can be designed to maximize the efficacy of cytotoxicity while minimizing risks to the patient. However, to our knowledge, no models have addressed how the effects of EMT alter interactions between the immune system and cancer, and the subsequent implications for treatment.

Here we develop a model with the goal of studying interactions between the tumor, the immune system, and EMT. We seek to describe a set of crucial molecular and cellular interactions in epithelial tumor cells (ETCs), including effects owing to DNA damage and mutation, to investigate the probability that in situ tumors will progress and, if so, when. A recent model of cancer–immune interactions^9^ described the effects of the TME on the risk of cancer, and we build on the core cell cycle component of this model, adding new interactions to the immune component of the model (which was previously modeled by a single interaction), as well as adding the effects of EMT. In doing so, we shift the focus of the previous model from cancer initiation to cancer progression. We do this to reflect the fact that cancer progression hinges on escape from the immune system and the fact that EMT has a more well-defined role during progression and metastasis. We seek to understand whether this more complex immune module will change our understanding of inflammatory effects on the tumor, and how the epithelial–mesenchymal axis influences these.

To test the model, we perform high-throughput analyses of data from The Cancer Genome Atlas (TCGA)^47^, using two clinical endpoints: the overall survival (OS) and the disease-free interval (DFI)^48^. To test model predictions on the effects of mesenchymal cell properties, we take a two-pronged approach: test whether EMT and inflammation can jointly separate clinical cohorts for a selection of carcinoma sub-types; then for those sub-types identified, we predict which genes regulate properties of tumor invasiveness. In the latter part, we find multiple lines of evidence that our model predictions agree with the literature, and make a number of new predictions. Following guidelines^48^, we investigated 14 TCGA tumor types recommended for analysis of the DFI endpoint.

In the next section, we analyze the general properties of the model, and we rigorously assess model behaviors via global “*one-at-a-time*” sensitivity analysis, which identifies parameters that are crucial for progression. We study these in more depth, focusing on the competing effects of EMT and of the immune system on progression, and discover that EMT intricately regulates progression: under certain regimes, a careful balance of EMT- and immune-driven processes can prolong invasion-free survival. We then test these predictions with a data analysis pipeline we develop using TCGA. We find strong evidence for the synergistic effects of inflammation and EMT for patients with bladder and uterine cancers.

## Results

### A multiscale agent-based model of EMT-immune-tumor cell interactions to study tumor progression

**Fig. 1:**
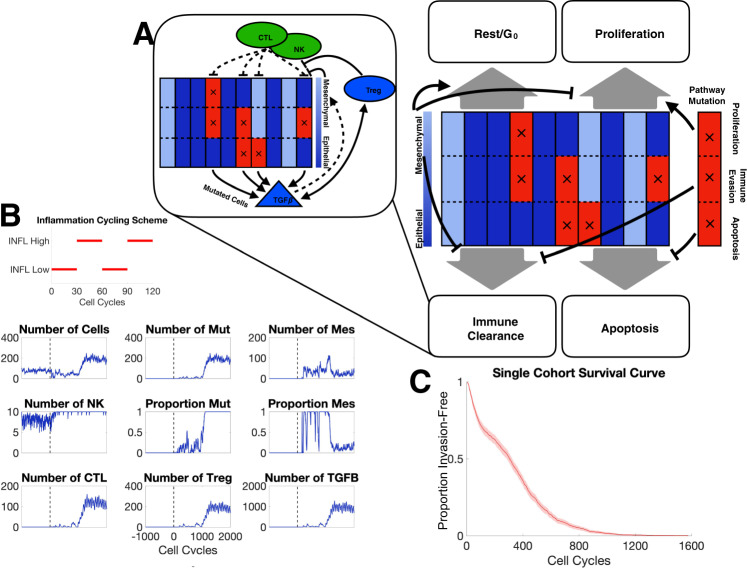
Overview of model structure and simulation outputs. **A** Schematic depiction of agent-based model components; each of the 10 columns represents one tumor cell divided into three compartments representing the state of three pathways with tumorigenic potential; blue/red denotes baseline/altered pathway activity. Black arrows depict cell fate regulation in each cell cycle. Inset depicts major interactions between the immune system and tumor cells. **B** A representative simulation of one patient. The parameter values used can be found in Table S1. The inflammation cycling scheme (red) is shown above the patient dynamics. The vertical dashed line denotes the end of the warmup period. *Mut* malignant cells, *Mes* mesenchymal cells. **C** Survival curve for one cohort of patients for parameter values given in Table S1.

We begin by investigating general features of the model to establish baseline conditions and assess the impact of different model components on the key measured outcomes: the probability of progression, and the time to invasion. During the cell cycle, cell fate is determined by rules that are influenced by EMT and immune interactions (Fig. 1A), e.g., if a cell undergoes EMT, its probability of proliferation is reduced; if it gains a mutation in the apoptosis pathway, its probability of apoptosis is reduced. Meanwhile, NK cells and CTLs attempt to clear malignant tumor cells, and deactivate upon successful tumor cell clearance; Tregs inhibit this cytotoxic activity (Fig. 1A Inset).

The inflammation cycling scheme for a typical in silico patient consists of alternating high and low regimes with corresponding effects on the cell populations (Fig. 1B). For this patient, after warmup, mutations are observed at a rate low enough that they are cleared by cytotoxic cells for ~700 cell cycles, after which the mutated and thus invasive cell population begins to grow, leading to large recruitment of CTLs and Tregs and a peak in the concentration of TGF-β. After 841 cell cycles, the proportion of invasive cells reaches 50%: the threshold defining progression, thus this patient has a time to the invasion of 841 cell cycles or 631 days. Beyond this time point, we see a rapid increase in the number of invasive cells until it comprises 100% of the tumor population. Interesting EMT dynamics are also observed, the proportion of MTCs peaks shortly after the tumor becomes invasive, subsequently, the majority of cells transition back to an epithelial state. We observe that although the NK population varies little over the simulation, CTLs and Tregs both undergo large expansions. CTLs and Tregs also appear to oscillate, however, note that this is a direct result of the inflammation state, and is not immune cell-intrinsic.

In order to quantify patient dynamics and invasion-free survival at a population level, we simulate large cohorts of patients similar to the single patient shown in Fig. 1B. For a cohort of 500 patients, we simulate survival curves and see that large number progress quickly to form invasive tumors, whereas a few lie in the tail of the distribution after the mutagenic event that a large number of tumors quickly progress while others take some time before progressing Fig. 1C. By ~1200 cell cycles (2.5 years), all tumors have become invasive.

### Global sensitivity analysis identifies a hierarchy of parameters in terms of their impact on model outcomes

Exploring the parameter spaces of systems biology models adequately is—in general—a hard problem. Fitting parameters via (Bayesian) parameter inference is advisable wherever possible^49^. Here, despite a wealth of data on tumor growth dynamics, a lack of sufficient molecular measurements (i.e., immune cell dynamics) precludes inference of the full model. In addition, although inference schemes for agent-based models are developing^50,51^, simulation times remain a hurdle^52^. Parameters for some components of the model studied previously can be constrained^9^, however, to characterize the full parameter space including new elements of our model here we use sensitivity analysis.

**Fig. 2:**
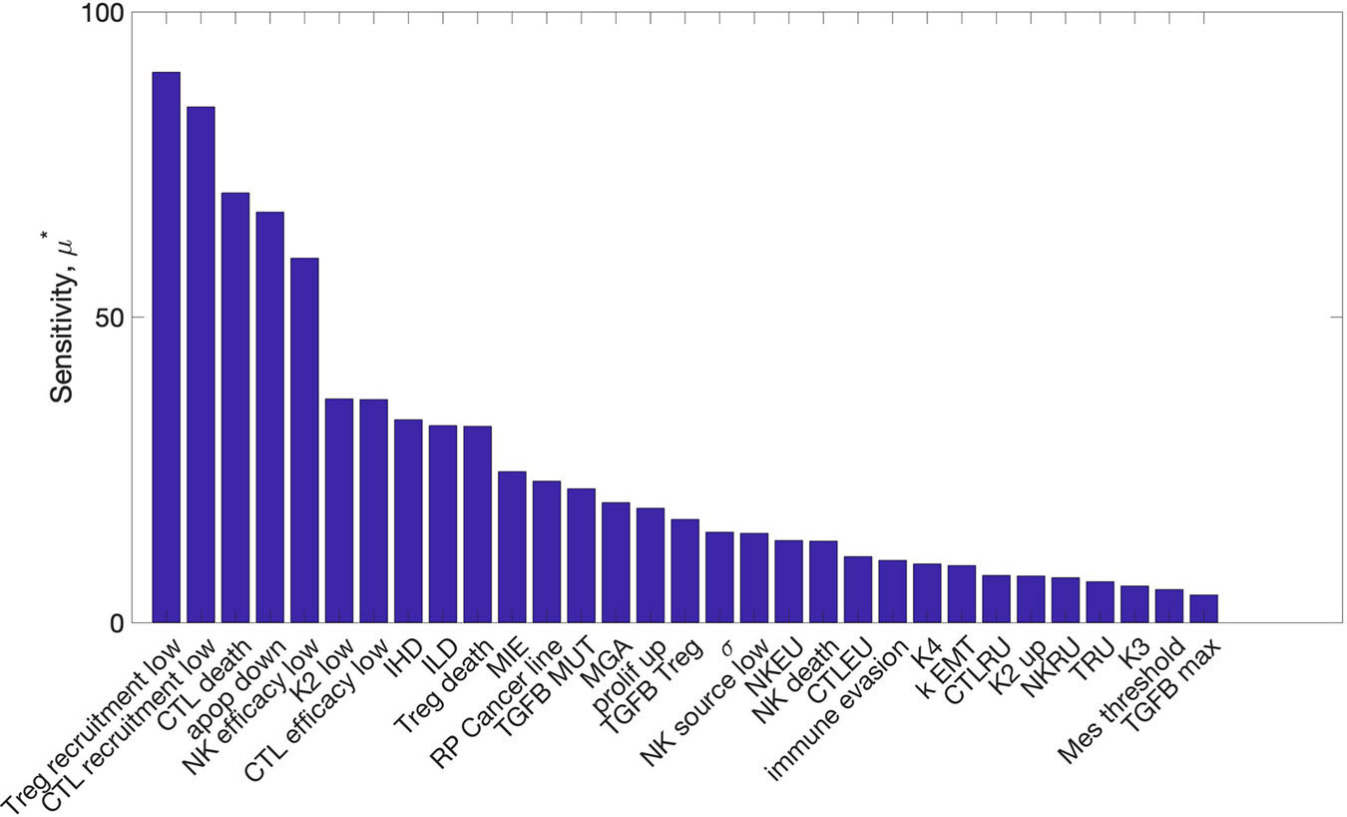
Global sensitivity analysis of model parameters. The sensitivity (μ*) denotes the average absolute change in the time to invasion over the range of variation of the parameter.

The results of Morris one-step-at-a-time sensitivity analysis on the 31 model parameters (Fig. 2) find a subset of parameters with much higher levels of sensitivity than others. The two most influential by this analysis are the recruitment rates of Tregs and CTLs in the low inflammation state. The parameters influencing EMT are also identified as influencing model outcomes. Since one goal of our analysis is to assess the specific effects of EMT on immune-cancer dynamics, parameters MIE and MGA are of particular interest. In addition, inflammation parameters controlling the duration of the high/low inflammation states (IHD and ILD) are of interest because they show moderate influence over model outcomes, and can readily be targeted by therapeutic treatments. For immune cell dynamics, the secretion of TGF-β by Tregs is found to be sensitive and thus will also be studied further below.

Notably, many of the most-sensitive parameters relate to the dynamics of immune cell populations; these prompt recommendations of experimental designs that would allow for reductions in the uncertainty in these parameters. In vitro, direct observation of ETC vs. MTC states is possible, and measurement of the relative clearance rates of ETCs vs MTCs by various immune components (NK cells, CTLs) would be informative. In vivo, although we may not be able to determine ETC vs MTC phenotypes at single-cell resolution, the efficacy of immune populations for tumor reduction, as well as (given its competing roles) the effects of TGF-β on tumor size would provide valuable information.

### Model predictions reveal that properties of the mesenchymal cell phenotype alter invasion-free survival times

**Fig. 3:**
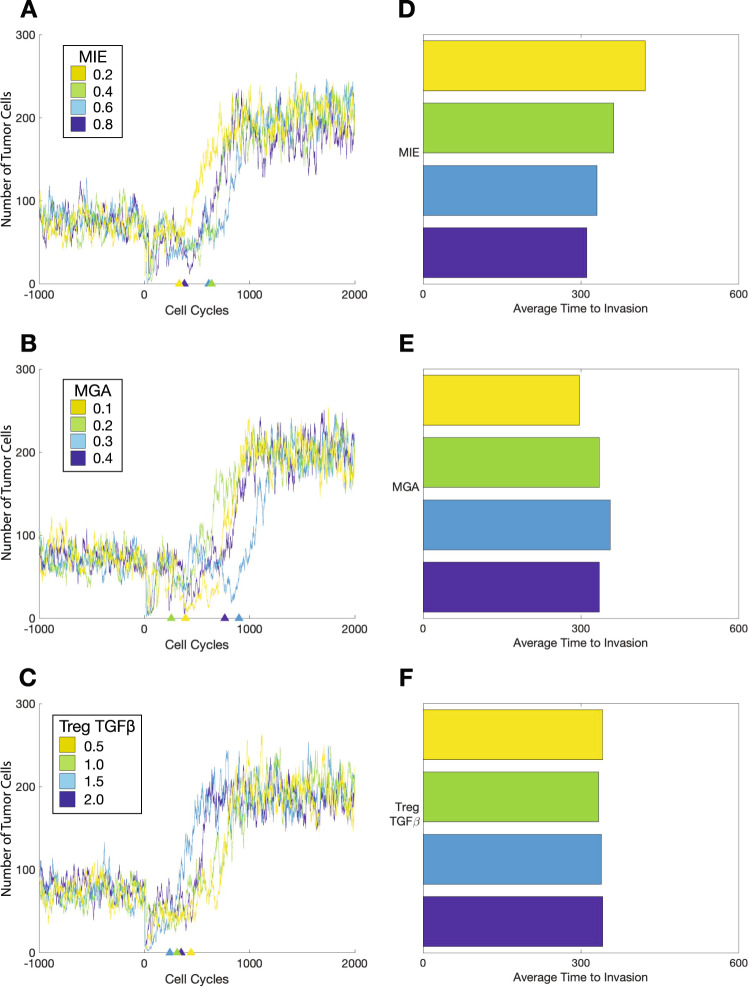
Effects of mesenchymal tumor cell properties on the time to invasion. Trajectories of one patient per cohort including warmup and 2000 cell cycles for **A** mesenchymal immune evasion (MIE); **B** mesenchymal growth arrest (MGA); **C** production of *TGF*-*β* by Tregs. **D** Average times to invasion for a patient cohort of 1000 for changes in MIE. **E** Average times to invasion for a patient cohort of 1000 for changes in MGA. **F** Average times to invasion for a patient cohort of 1000 for changes in Treg production of *TGF*-*β*.

To study the effects on EMT and MTC phenotypes on tumor dynamics, we analyzed the invasion-free survival times predicted by the model in response to changes in three model parameters: MIE, MGA, and the levels of TGF-β that are produced by Tregs. We varied each of these three parameters over a prior range, and for each parameter value, we simulated the model 1000 times, i.e., creating an in silico cohort of 1000 simulated patients, where simulated patients were censored after 2000 cell cycles. We then performed survival analysis for each cohort by computing the Kaplan–Meier (KM) curve associated with time to invasion. With all other parameters held constant, we studied the effects of MIE on invasion-free survival, varying MIE in the range (0.2, 0.8) (Fig. 3A). As MIE increases, the invasion-free survival decreases monotonically (Fig. 3D); i.e., as the subpopulation of invasive cells becomes more resistant to immune clearance, the tumor as a whole grows more resilient and thus can grow faster.

The relationship between MGA and invasion-free survival times exhibits a different trend. With all other parameters held constant, we studied the effects of MGA on invasion-free survival, varying MGA in the range [0.1, 0.4] (Fig 3B). We found that for smaller values of MGA, increasing MGA results in increasing the invasion-free survival, however for larger values, increasing MGA led to the invasion-free survival times decreasing (Fig 3E). This non-monotonic relationship is explored in greater detail below.

TGF-β varies according to its production by tumor cells and its production by Tregs. We assess the effects of varying the production of TGF-β by Tregs on invasion-free survival (Fig 3C, F), and find that at lower production rates of TGF-β, the survival curve initially declines faster, whereas higher production rates result in a steeper drop off in survival later. Although lower values of TGF-β production lead to a steeper initial decline, these differences vanish for higher values of TGF-β. The steeper initial decline may be owing to the rapid clearance of tumor cells by adaptive immune cells before Tregs have had sufficient time to modulate the TME through the secretion of TGF-β.

### Analysis of simulation outcomes identifies a key EMT regime that maximizes invasion-free survival times

**Fig. 4:**
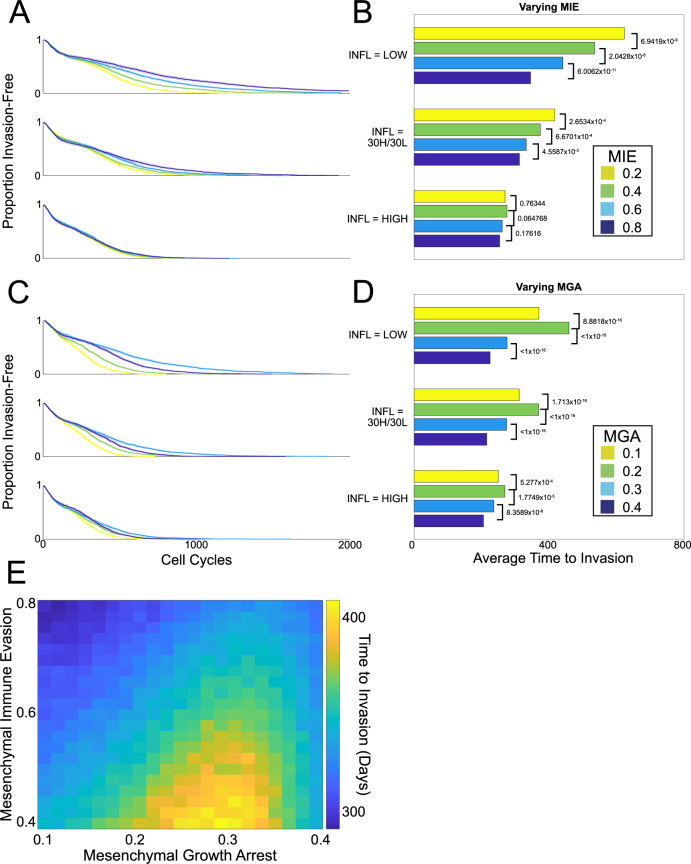
Effects of inflammation on the time to invasion under different cycling schemes. **A**, **B** As MIE varies, survival curves (each of 200 patients) and corresponding bar plots to summarize the mean time to invasion for each cohort are shown. *P* values for log-rank tests on the corresponding survival curves are shown. **C**, **D**. As MGA varies, survival curves and corresponding bar plots to summarize the mean time to invasion for each cohort are shown. *P* values for log-rank tests on the corresponding survival curves are shown. **E** Summary of the effects of MIE and MGA on invasion-free survival.

To investigate how competing interactions within the inflammatory TME affect EMT, we explored the effects of varying inflammation on invasion-free survival. Model simulations were used to create in silico patient cohorts for different inflammation conditions. Three conditions were compared: permanently low inflammation; permanently high inflammation; or variable (periodic high/low) inflammation. For each inflammatory condition, we varied the mesenchymal parameters (see Fig. 4), and for each choice of parameters, we simulated 1000 patients, censoring them after 2000 cell cycles. We then performed survival analysis by computing the KM curves associated with time to invasion. Compared with the other inflammation states, permanently high inflammation results in less-variable outcomes with respect to differences in mesenchymal parameters (Fig. 4). When inflammation was either permanently or temporarily in the low state, the invasion-free survival time was negatively correlated with MIE (Fig. 4A, B). For varying MGA, the invasion-free survival varied non-monotonically (Fig. 4C, D). For each inflammation condition, a local maximum was found with respect to MGA, with a peak close to Δ_MGA_ = 0.2.

These differences in the mean invasion-free survival times lead to predicted variation in clinical outcomes: tumors are contained in situ for up to twice as long as they would have been otherwise owing to variation in the rates of MGA. This points to possible therapeutic outcomes: the model predicts that a patient experiencing intermittent high inflammatory attacks will benefit directly from EMT-directed therapies, however, patients for whom a relatively high inflammation state is observed chronically will not obtain this benefit.

In contrast, when MIE is varied under different inflammation cycling schemes, for all the conditions studied, the model predicts that increasing MIE will decrease the invasion-free survival (i.e., worsen cancer progression and prognosis). Thus, reductions in MIE will lead to improvements in patient outcomes. To summarize the mesenchymal properties of immune evasion and growth arrest, we plot the joint density of these parameters against the time to invasion (Fig. 4E). We see that for a given value of MIE, there is a value of MGA that maximizes the time to invasion.

Testing this prediction in vivo is challenging since the definition of MGA is based on tissue-culture assays^23^, the equivalent of which is unavailable in animal models. Furthermore, the unimpeded cancer dynamics of progression that the model simulates are clearly at odds with clinical practice. The model also assumes precisely known onset of tumorigenesis—not available in experimental or clinical models, and is confounded by tissue-specific variation^53^. Thus, to assess mesenchymal phenotype-associated model predictions against data, we resort to the use of gene expression profiles across a range of tumors, available through the cancer genome atlas.

### Model predictions on the influence of mesenchymal phenotypes on clinical outcomes are supported by TCGA data analysis

To test the prediction that MGA rates exert essential control on invasion-free survival times (Fig. 4E), we performed an analysis of 14 cancer types from TCGA and assessed the importance of mesenchymal proliferation-associated genes against clinical outcomes. In order to do so, we must first connect clinical outcomes with EMT-related phenotypes. This can be achieved using either individual genes or gene signatures, e.g., via gene ontology (GO) terms^54,55^. We chose to take an unbiased approach (see Methods): first, to identify relevant cancer types, we studied whether GO terms were statistically associated with survival; and second, from this set of cancer types and GO terms, we exhaustively searched all gene pairs for their (joint) impact on the DFI as means to study how joint changes in immune- and EMT-related processes impacted patient outcomes (Supplementary Fig. S5).

Using OS as an endpoint, and applying strict significance thresholds (see Supplementary Methods Section 3.1), we found that for three cancer types, EMT-inflammation-associated genes predicted clear differences between patient groups. The three significant tumor types were bladder (BLCA), uterine (UCEC), and liver cancer (LIHC) (Supplementary Figs. S6–S8).

Using DFI as an endpoint, we modeled the relative effects of EMT-inflammation-associated genes by clustering patients from each tumor type into two groups (high or low) based on their DFI (see Methods); these data contain 184 patients for BLCA, 114 patients for UCEC, and 311 patients for LIHC. For this clustering, the predictive accuracies (obtained by leave-one-out cross-validation) were 0.68 (BLCA), 0.69 (UCEC), and 0.621 (LIHC) (Supplementary Figs. S9–S11). Several of the other 11 cancer types tested also displayed mesenchymal proliferation-associated effects, however, these cancers were filtered out at the previous step, as they did not meet the significance thresholds set above.

**Fig. 5:**
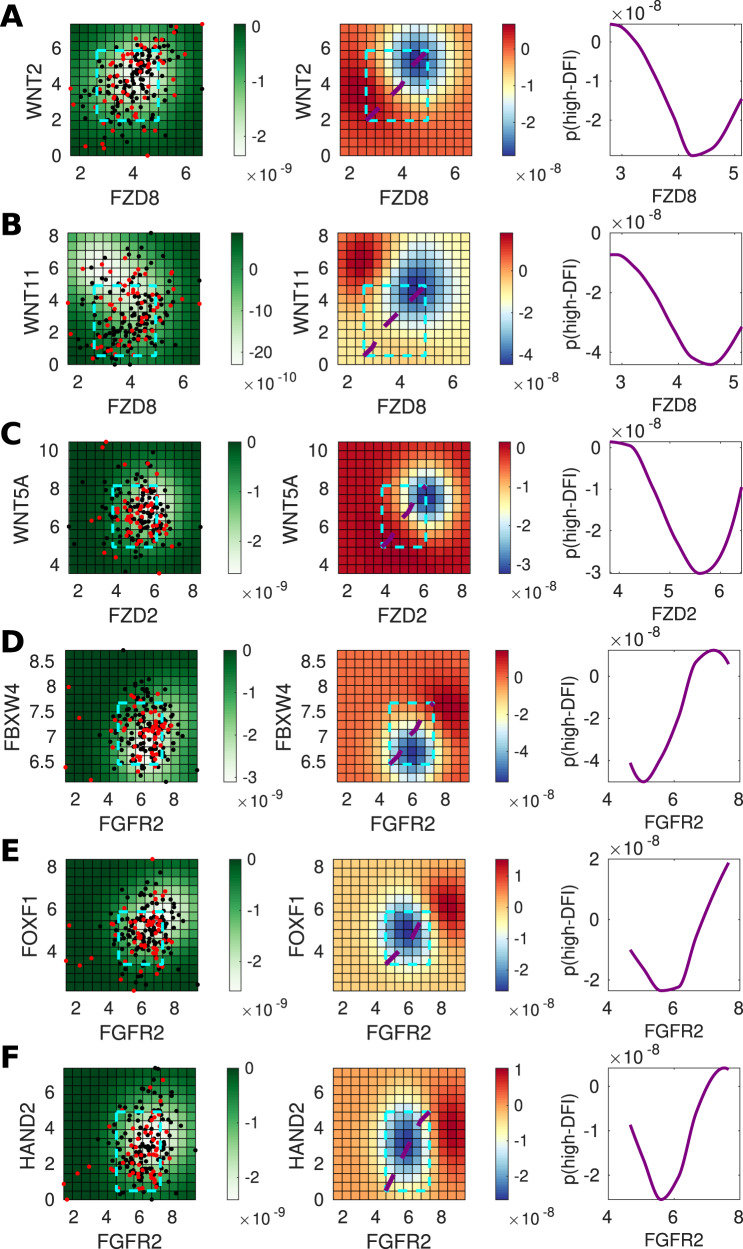
Genes predictive of invasiveness in BLCA. **A** For gene pair *WNT2* and *FZD8*, the left panel shows the posterior variance on log–log expression plot of the predicted probability overlaid with patient samples (red = low-DFI, black = high-DFI), 90% confidence interval box drawn for standardized expression values (cyan); middle panel: posterior log probability of high-DFI over the same region as left, where the diagonal line (purple) shows the co-expression trend (diagonal line through the 90% CI of standardized expression values); right panel: posterior log probability of high-DFI plotted against the expression of *FZD8*, values simulated along the diagonal (purple) corresponding to the middle panel. **B** As above for *WNT11* and *FZD8*. **C** As above for *WNT5A* and *FZD2*. **D** As above for *FBXW4* and *FGFR2*. **E** As above for *FOXF1* and *FGFR2*. **F** As above for *HAND2* and *FGFR2*.

**Fig. 6:**
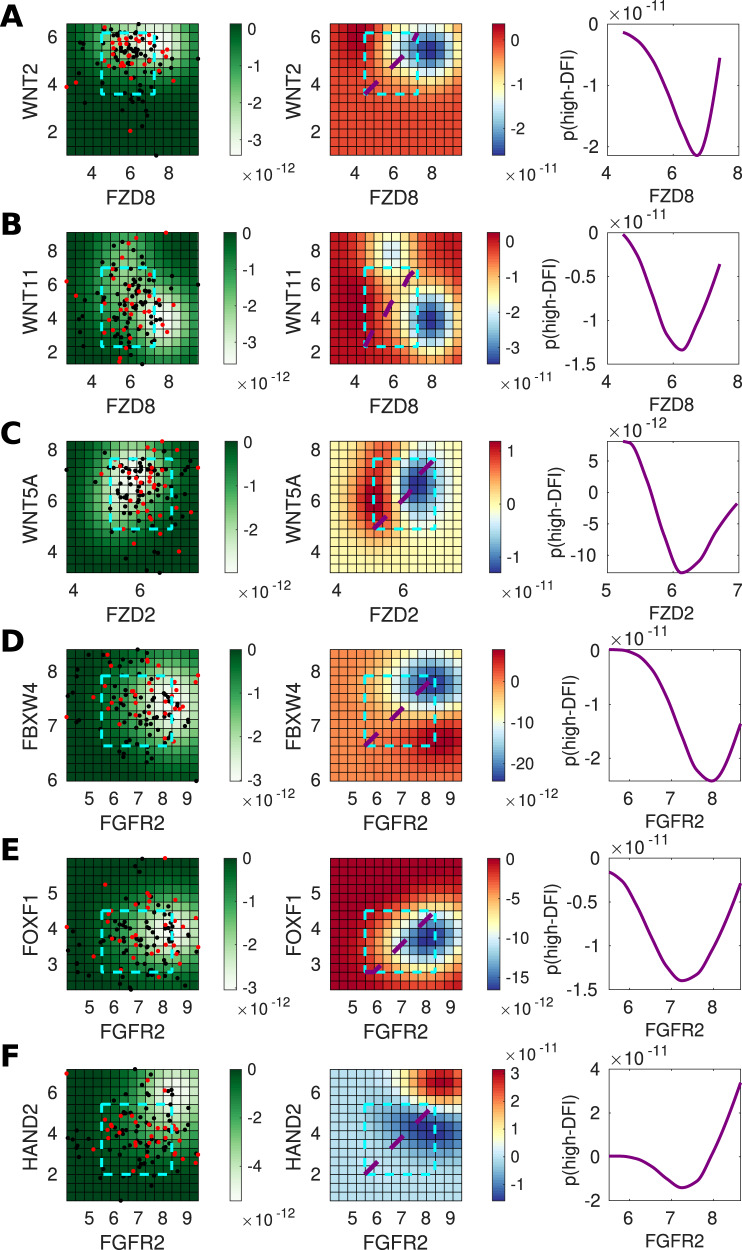
Genes predictive of invasiveness in UCEC. **A** For gene pair *WNT2* and *FZD8*, the left panel shows the posterior variance on log–log expression plot of the predicted probability overlaid with patient samples (red = low-DFI, black = high-DFI), 90% confidence interval box drawn for standardized expression values (cyan); middle panel: posterior log probability of high-DFI over the same region as left, where the diagonal line (purple) shows the co-expression trend (diagonal line through the 90% CI of standardized expression values); right panel: posterior log probability of high-DFI plotted against the expression of *FZD8*, values simulated along the diagonal (purple) corresponding to the middle panel. **B** As above for *WNT11* and *FZD8*. **C** As above for *WNT5A* and *FZD2*. **D** As above for *FBXW4* and *FGFR2*. **E** As above for *FOXF1* and *FGFR2*. **F** As above for *HAND2* and *FGFR2*.

We used Gaussian process classification to identify relationships between mesenchymal proliferation genes based on their ability to predict invasiveness (high or low-DFI). We focus on interactions within the TGF-β and Wnt pathways, given their important roles in mediating EMT^56^, and regulating cancer stem cell identity^57,58^. TGF-β and Wnt pathways interact at multiple points, including through the *LEF1/TCF* complex^59^, and via dimerization of their respective membrane-bound receptors^60^. We found that for both canonical and non-canonical Wnt signaling, higher levels of signaling lead to worse outcomes (Figs. 5A–C and 6A–C), in agreement with the literature^57,58^. The prediction for each gene pair is summarized in the right-hand column: a slice through the co-expression plot (purple line in middle column) shows that as the co-expression of the Wnt ligand and its receptor increases, the probability of a high-DFI (better outcome) decreases. This is seen consistent across Wnt ligand–receptor gene pairs, with the exception in UCEC of *WNT11* and *FZD8* (Fig. 6B), where the co-expression effects are less clear. Overall, these predictions agree with expected tumorigenic roles for canonical^61,62^ and non-canonical^60,63,64^ Wnt signaling in the bladder (BLCA) and uterine (UCEC) cancers.

Several other gene pairs also predicted differences between high vs low-DFI patient groups (Figs. 5D–F and 6D–F). For the gene pairs (*FGFR2*, *FBXW4*) and (*FGFR2*, *FOXF1*), the joint distributions of these gene pairs showed unimodal peaks, thus recapitulates the distribution of MGA that was produced through model simulations (Fig. 4E). We see that in independent TCGA data analysis, as predicted in the model, there exists a “goldilocks” region with respect to the MGA of the mesenchymal phenotype that most benefits invasion-free survival.

The TCGA data analysis led to further predictions regarding the effects of gene co-expression on patient outcomes. In Fig. 5D (middle panel), we show that a tumor-suppressor effect of *FGFR2* in bladder cancer is predicted by our model. Although FGF signaling plays opposing roles in cancer, and FGFs can be upregulated in tumors relying on FGF signaling for growth^65^, *FGFR2* is implicated as a tumor suppressor in prostate and bladder cancer^66,67^. We also predict a suppressive role for *FBXW4* (Fig. 5D, middle panel): for given *FGFR2* expression, increasing *FBXW4* leads to better outcomes. This agrees with literature suggesting that *FBXW4* is lost or mutated in almost 40% of urinary tract cancers^68^. This analysis predicted that *FGFR2* and *FBXW4* act synergistically in BLCA, such that higher expression levels of both lead to greater outcomes than the high expression of either gene alone (5D, middle and right panels). In comparison, for UCEC, the role is less clear, although the tumorigenic effect of *FGFR2* in uterine cancer is evident at high levels of *FBXW4* (6D), in line with previous studies reporting mutations that provide constitutive activation of *FGFR2* in a subset of endometrial cancer^69^.

For BLCA, our analysis predicts that high *FGFR2* and *FOXF1* co-expression will improve patient outcomes (Fig. 5E). The tumor suppressor *FOXF1* is a p53 target and it is epigenetically silenced in breast cancer^70,71^, however, to our knowledge no previous tumor-suppressive role for it has been reported in BLCA, either alone or co-expressed with *FGFR2*. This effect is not seen for UCEC (Fig. 6E), where our analysis predicts that the effects of *FGFR2* are tumorigenic in this region, but not affected by *FOXF1* expression (i.e., no significant differences between high and low co-expression). We also predict that high co-expression of *FGFR2* and *HAND2* improves outcomes in UCEC (Fig. 6F); in contrast to the effects seen for the co-expression of *FGFR2* with either *FBXW4* or *FOXF1* (Fig. 6D, E), where, in each case, higher *FGFR2* expression led to worse outcomes. *HAND2* antagonizes FGF-dependent epithelial cell proliferation and is a critical regulatory component of both healthy and cancerous endometrial proliferation^72,73^. For BLCA, we observe a less pronounced although still suppressive effect owing to *HAND2* expression (Fig. 5F), in line with the previous reports^74^.

## Discussion

Despite the importance of interactions between cancer and the immune system, as well as the role of EMT in cancer, to the best of our knowledge, no model has previously combined these three components. We saw this as a particularly pressing need given the shared factors influencing all these components, including TGF-β and Wnt signaling. We developed an agent-based model to study cancer, the immune system, and EMT, during the progression from in situ tumor to invasive disease. The model predicted mesenchymal growth rate as a crucial parameter in determining invasion-free survival. Via TCGA data analysis, we studied the effects of mesenchymal phenotype-associated genes on patient outcomes and found that EMT-associated genes worsened prognosis, in agreement with the predictions of the model. We strived to constrain model complexity wherever possible, for interpretability, and so that the model is of use to make biological statements. Nonetheless, the solutions of the model that were obtained through simulation rely crucially on the assumptions that were made during model construction. As data were not available for model fitting and parameter inference, the extent to which model simulations reflect the underlying biology remains to be carefully quantified. Below, we discuss the limitations of this model and describe the future work that will be needed to overcome them.

We found that the model recapitulated carcinoma dynamics. Parameter sensitivity analysis identified model parameters exerting key control over model behavior. Focusing on these led us to identify that increasing MIE and increasing Treg TGF-β production both lead to shorter invasion-free survival times. However, varying the level of inflammation led to paradoxical effects with regards to MGA: under regimes with periods of low inflammation, an optimal level of MGA can improve outcomes and maximize invasion-free survival. That EMT alters the dynamics of cancer progression is well-established^14,75^; here, we are able to identify particular properties of the mesenchymal phenotype responsible.

To capture the essential characteristics of the model, we summarized model simulation of in silico patient studies with a single parameter: the invasion-free survival time. There are, of course, many trajectories that result in progression to invasion. Further analysis of the transient cell dynamics in tumors during cancer progression is needed to gain insight into the EMT-associated dynamics. A strong assumption of the model is that all cells are well-mixed, i.e., we do not take into account spatial effects. Although these are of course crucial, it is important to first characterize EMT-immune–tumor interactions in a well-mixed system, to define a baseline. An important extension of the model will be to include spatial interactions among tumor and immune cells.

We set out to study phenomena resulting from tumor–immune–EMT interactions, and the model we developed predicted that a specific property of the mesenchymal phenotype—the MGA—exerted key control over tumor invasiveness. In order to test this prediction, we employed a data analysis framework using gene expression data from TCGA. In support of the model prediction, we found that mesenchymal-associated genes controlled outcomes and predicted differences between high vs. low-DFI analysis yielded predictions of the effects of single genes or gene pairs, many of which corresponded to known effects, including the effects of both canonical and non-canonical Wnt signaling on tumor progression. Our modeling also predicted opposing roles for FGF signaling in the bladder and uterine cancers: where *FGFR2* exerts a tumor-suppressor effect in bladder cancer yet a tumorigenic effect in uterine cancer. Evidence for these opposing roles already exists in the literature, but notably, through our modeling, we also predict entirely novel interactions between *FGFR2* and other transcription factors (*FBXW4, FOXF1*, and *HAND2*) that act to enhance or suppress the effects of *FGFR2* alone, and could offer novel therapeutic strategies.

In future work, further development of the inflammation module is important given the large and at times paradoxical roles that the inflammatory state exerts on tumor cells and invasion-free survival. At present, inflammation is modeled as cycling between high and low schemes of variable duration, independent of other model components. Yet, several known factors contribute to the inflammatory state. For example, model extensions could assume that the level of inflammation depends on the number of and the degree of mutations that tumor cells harbor. The competing effects that TGF-β exerts on the tumor and its microenvironment also warrant further investigation. We found that—below a certain threshold—reduction of TGF-β increases the time to invasion, i.e., reducing TGF-β in the TME benefits survival. Experimental work in support of this result includes a study of TGF-β tumor suppression in pancreatic cancer through the promotion of EMT^76^. The TGF-β pathway is, however, implicated in numerous other cellular signaling processes besides EMT; changing TGF-β concentration even in a local environment could have large off-target effects. Indeed, it has been shown that TGF-β promotes invasion and heterogeneity while suppressing cell proliferation in squamous cell carcinoma^77^. To account for this complex signaling, future work should incorporate the effects of signaling factors downstream of TGF-β on the cancer dynamics. It is also important to note that in this model, EMT is initiated entirely by TGF-β. Although TGF-β does play a large role in cancer EMT, it is by no means the only factor at play; in reality, cells must contend with and respond to a milieu of EMT-associated signals. There are also other cells in the TME that can express mesenchymal markers, most notably cancer-associated fibroblasts^78^, thus presenting a possible confounding variable in TCGA gene expression analysis; future analyses of single-cell data sets will help to deconvolute these sources.

Tumor heterogeneity often helps the tumor to evade immune effects and complicates our approaches to treatment. A rigorous study of the consequences of the increased heterogeneity that follows disease incidence (i.e., decanalization^79^) is too often sidelined, despite mounting evidence in support of its prominent role in cancer evolution^80–82^. Despite these challenges, great progress in predicting disease complexity continues to be made. As we are rapidly approaching a new generation of immunotherapies, it is these very complexities that we must better understand in order to control or eradicate the disease.

## Methods

We develop an evolutionary agent-based model to describe the relationships between cancer, the immun e system, and EMT, building on the cell cycle and tissue-cell components described in ref. ^9^. We use simulation and sensitivity analysis to analyze the model, and compare model outputs with clinical outcomes through TCGA data analysis.

In the agent-based model, agents are cells that initially comprise an in situ tumor, i.e., one that exhibits no invasive properties. During model simulation, tumor cells can acquire mutations altering one or more of three key pathways (Fig. 1A), beginning a progression towards invasiveness. EMT affects tumor growth dynamics: we model tumor cells as in one of two states: epithelial tumor cells (ETCs) and MTCs and permit transitions between these states (we leave the addition of intermediate EMT states as future work^83,84^). Tumor cells—along with their individual mutational profiles and EMT status—are the central variables in the model. In addition, three types of immune cells—NK cells, CTLs, and Tregs—are included as continuous variables. In response to the recognition of neoantigens, these populations engage in a set of interactions with one another and with the tumor, shaping the disease trajectory. The local inflammatory conditions of the TME in general, and the concentration of TGF-β, in particular, are included as modulators of the immune system and of tumor–immune interactions, including induction of EMT. An overview of equations governing the mode is given in Table 1, and all parameter values used along with their descriptions can be found in Supplementary Table S1. The procedure to simulate the model is given by pseudocode in Algorithm 1.

**Table 1:** Description of the model.

Component	Variable	Type	Governing equations
Tumor cells	*N*_*C*_	Agent-based	$\kern1.8pc\displaystyle{\rho }_{P}=p(1+{\delta }_{P}{{{\Delta }}}_{P})(1-\zeta {{{\Delta }}}_{{{{{\rm{MGA}}}}}})\frac{1}{1+{N}_{C}/{K}_{0}}\kern6.1pc (1{{{\mbox{a}}}})$ $\kern1.8pc {\rho }_{A}=d_{C}(1-{\delta }_{A}{\Delta }_{A})\kern13.5pc(1{{{\mbox{b}}}})$ $\kern1pc\displaystyle{\rho }_{{{{{\rm{NK}}}}}}={\delta }_{{{{{\rm{MUT}}}}}}\frac{{N}_{{{{{\rm{NK}}}}}}}{\frac{{N}_{C}}{{K}_{1}}+{N}_{{{{{\rm{NK}}}}}}}\frac{{E}_{{{{{\rm{NK}}}}}}}{1+\frac{{N}_{{{{{\rm{Treg}}}}}}}{{K}_{2}}}(1-{\delta }_{{{{{\rm{IE}}}}}}{{{\Delta }}}_{{{{{\rm{IE}}}}}})(1-\zeta {{{\Delta }}}_{{{{{\rm{MIE}}}}}})\kern3pc (1{{{\mbox{c}}}})$ $\kern1.8pc\displaystyle{\rho }_{R}=1+\zeta p(1+{\delta }_{P}{{{\Delta }}}_{P}){{{\Delta }}}_{{{{{\rm{MGA}}}}}}\frac{1}{1+{N}_{C}/{K}_{0}}\kern6.6pc(1{{{\mbox{d}}}})$
NK cells	*N*_*NK*_	ODE	$\kern0.8pc\displaystyle{N}_{\,{{{{\rm{NK}}}}}}^{\prime}={\sigma }_{{{{{\rm{NK}}}}}}-{d}_{{{{{\rm{NK}}}}}}{N}_{{{{{\rm{NK}}}}}}\kern13.45pc(2)$
CTLs	*N*_*CTL*_	ODE	$\kern0.5pc\displaystyle{N}_{\,{{{{\rm{CTL}}}}}}^{\prime}={\sigma }_{{{{{\rm{CTL}}}}}}{N}_{{{{{\rm{MUT}}}}}}^{* }-{d}_{{{{{\rm{CTL}}}}}}{N}_{{{{{\rm{CTL}}}}}}\kern11.05pc(3)$
Tregs	*N*_*Treg*_	ODE	$\kern0.4pc\displaystyle{N}_{\,{{{{\rm{Treg}}}}}}^{\prime}={\sigma }_{{{{{\rm{Treg}}}}}}{N}_{{{{{\rm{MUT}}}}}}^{* }\frac{\tau }{1+\tau /{K}_{4}}-{d}_{{{{{\rm{Treg}}}}}}{N}_{{{{{\rm{Treg}}}}}}\kern7.48pc(4)$
TGF-*β*	*τ*	Algebraic	*τ* = *τ*_MUT_*N*_MUT_ + *τ*_Treg_*N*_Treg_(5)

Tumor cells (*N*_*C*_) are modeled discretely as agents; all other variables are modeled continuously. Each tumor cell exists in one of 2^3^ states by mutation profile, see Methods for description.

### Model development: definition of model states and parameters

We developed an agent-based model of tumor evolution consisting of three interacting dynamic components: the tumor cells; the immune system; and the role of EMT.

#### Tumor cell dynamics and evolution

At each time step, tumor cell fate decisions are made according to a set of probabilities given in Eq. (1) (Table 1). These define the probability that a tumor cell will undergo proliferation (*ρ*_*P*_), apoptosis (*ρ*_*A*_), clearance by NK cells (*ρ*_*NK*_), or rest in *G*_*O*_ (*ρ*_*R*_).

Following cell fate updates, the mutational signature of each cell is updated. To define the signature, we consider three key phenotypes, as in^9^. The “proliferation” mutation increases the probability of the cell exiting *G*_*o*_ to proliferate; the “apoptosis” mutation decreases the probability of a cell undergoing apoptosis; and the “immune evasion” mutation decreases the probability that a mutated cell will be cleared by immune cells. Initially, tumor cells do not harbor any of these mutations, which are acquired during the simulation and faithfully passed on to daughter cells. For a given cell, *δ*_*P*_, *δ*_*A*_, and *δ*_IE_ are boolean values that indicate if the cell has the proliferation, apoptosis, or immune evasion pathway mutations, respectively.

#### Immune system dynamics and inflammatory states

The immune system is modeled by three immune cell types: NKs, CTLs, and Tregs. The population sizes of these three components are given *N*_*NK*_, *N*_*CTL*_, and *N*_*Treg*_, respectively, and their dynamics are given in Eqs. (2–4) in Table 1. NKs, part of the innate immune response, is not affected by tumor growth but can clear tumor cells. CTLs, part of the adaptive immune response, also clear tumor cells (with greater efficiency than NK cells), and are recruited to the tumor in response to its growth. Upon tumor cell clearance, the respective NKs and CTLs are deactivated and removed from the total immune populations. Tregs act to suppress the function of NKs and CTLs. In addition, Tregs release TGF-β (defined in the model by *τ*), which increases the probability of EMT.

Inflammation is modeled as a cycling scheme between low and high inflammatory states, as previously modeled^9^, with varying inflammation high and low durations, controlled by the parameters IHD and ILD, respectively. The inflammatory scheme is fixed for the entirety of the simulation. At the preset times at which the inflammatory state switches (low to high, or high to low), the immune activity parameters are updated according to the values given in Table S1.

#### The role of EMT on tumor dynamics

Each tumor cell exists in either an epithelial or mesenchymal state. (partial EMT, while playing essential roles, is outside of the model scope considered here.) Cells are modeled by a continuous EMT score between 0 and 1, which is dependent upon its past state and the external TGF-β concentration (Eq. (6) in the Supplementary Text). If the EMT score is above a fixed threshold (given by *T*_*MES*_, Table S1) in a given cell cycle, the cell undergoes EMT and is assigned as a MTC; otherwise, it becomes/remains an ETC. The boolean parameter *ζ* indicates the cell fate: mesenchymal (true) or epithelial (false). When a cell undergoes EMT or the reverse (MET), its responses to the TME are affected. MTCs experience a decrease in proliferation probability relative to ETCs (MGA) and an increase in immune evasiveness (MIE). The parameters that control these changes are Δ_MGA_ and Δ_MIE_. These parameters are both bounded on the interval [0, 1] and represent the proportion change from the epithelial state. That is, Δ_MGA_ = 0 corresponds to no reduction in proliferation for MTCs and Δ_MGA_ = 1 to a complete reduction, i.e., no proliferation of MTCs. For Δ_MIE_, a value of 0 indicates no additional evasion of the immune system and a value of 1 indicates complete evasion of the immune system, i.e., MTCs will never be cleared by immune cells.

### Model development: algorithmic implementation and simulation

#### Initializing the model

Simulations are initialized with *N*_0_ in situ tumor cells. Given that different parameters can give rise to different steady states, we simulate a warmup period consisting of a fixed number of cycles where no mutations occur and only NK cells are present (Algorithm 1). This allows the tumor to complete its exponential growth phase and reach carrying capacity. We determined that the tumor carrying capacity was reliably reached after 1000 cell cycles of warmup. We then begin all in silico experiments from these post-warmup conditions. After the warmup period, mutations are permitted and the adaptive immune response (CTL and Treg populations) is turned on. Thus, any changes to the tumor after warmup are owing to its evolving mutational profile and simultaneous changes in the surrounding immune conditions.

##### Algorithm 1

Simulation of one tumor

**Result:** Determine time to progression

initialization;

**for** *Pre-set number of warmup cycles* **do**

Assign and apply tumor cell fate decisions (Eq. (1));

Update NK population (recruitment, apoptosis, and exhaustion);

**end**

**while** *Proportion of mutant tumor cells* < 50% *of total* **do**

Assign and apply tumor cell fate decisions (Eq. (1));

**for** *Cells that proliferate* **do**

**if** *rand() < probability of mutation* **then**

Cell acquires new mutation; reset cell-autonomous mutation probability to 0;

**else**

Increase cell-autonomous mutation probability

**end**

**end**

Update immune population dynamics over time span of one cell cycle (Eq. (2)–(4));

Update TGF-*β* concentration (Eq. (5));

Update the EMT score for each tumor cell;

**end**

#### Simulation of tumor cell dynamics

Each tumor cell is updated once every 18 hours (the approximate length of one cell cycle). During each cycle, the fate of each cell is assigned, based on its probability of undergoing proliferation (*ρ*_*P*_), apoptosis (*ρ*_*A*_), immune clearance (*ρ*_*NK*_), or resting in *G*_0_ (*ρ*_*R*_), according to Eq. (1) in Table 1. The probability of proliferation is increased by a mutation occurring in the proliferation pathway and decreased if the cell is in a mesenchymal state. The probability of apoptosis varies according to the mutational profile of the cell. The probability of clearance by immune cells is affected by the number of mutations harbored: cells with one or more mutations are assumed to be more immunogenic and have a higher probability of being cleared by the immune system. Cells with mutations to immune evasion pathways, or which are in a mesenchymal state, are in a state of increased “immune evasiveness” and as a result, their probability of immune system clearance is reduced.

During every cell cycle, all tumor cells that proliferate have a probability of gaining a mutation in one of the three pathways investigated. This probability is cell-specific and changes over time obeying two simples rules: gaining a mutation resets this probability to 0; not gaining a mutation increases this probability by a fixed amount, 10^−4^. The EMT score is also updated at the end of every cell cycle, if applicable, causing cells to change fates from ETC to MTC, or vice versa.

#### Simulation of immune population dynamics

Once all tumor cells have been updated and fates chosen accordingly, non-tumor model components are updated. Immune cell populations are updated in two steps. First, immune cell exhaustion (via loss of efficacy or PD1 signaling) is calculated based on the number of tumor cells cleared. Second, immune cells (NKs, CTLs, Tregs) are updated according to a system of coupled ordinary differential equations that govern their population dynamics (See Table 1). CTL and Treg recruitment rates are dependent on the number of immune-cleared tumor cells, corresponding to their role in the adaptive immune system relying on antigen stimulation; in addition, TGF-β enhances the recruitment rate of Tregs. Finally, the concentration of TGF-β is updated; this depends on the dynamics of Tregs and—to a lesser extent—invasive tumor cells, as sources of TGF-β in the model.

### Model development: summary statistics and analysis of outcomes

#### Summary statistics for the progression to invasive disease

When the proportion of tumor cells that harbor at least one pathway mutation reaches 50% of the total tumor size, the tumor is defined as having progressed to an invasive state. The summary statistic tracked for all simulations is the “time to invasion,” i.e., the number of cell cycles until the tumor enters an invasive state, after warmup. If this threshold is never reached, the simulation ends when the maximum number of cycles is reached.

#### Global sensitivity analysis and parameter estimation

To study parameter sensitivity, we implemented Morris a one-step-at-a-time global sensitivity analysis. Parameters are varied one at a time from a set of sampled “base” points and the resulting simulations recorded^85,86^. For each run, we simulated 1000 patients and initialized the Morris sampling with 30 points in parameter space (at least 10 are recommended in ref. ^86^. Parameter sampling is a choice of prior parameter distributions. For many parameters, such as for the immune population dynamics, measurements or estimates were available from literature^87^. For parameters such as MIE and MGA related to the mesenchymal phenotype, little prior information was available, thus these were sampled across all possible values in [0, 1]. Tumor size in the model was scaled from cell numbers on the order of 10^9^ cells^87^ to the order of 10^2^, and parameter values were scaled accordingly. Where parameter estimates existed, the prior for parameter *θ*_*i*_ is given as *θ*_*i*_ ~ N(*m*_*e*_, 2*m*_*e*_), where *m*_*e*_ is the previous estimate and we take twice this value as the variance to obtain a range of samples that does not rely too heavily on previous work. The Morris algorithm computes the sensitivity, μ*, as the average of the absolute change of the output, which in our model is the area under the survival curve (Fig. 2).

### Analysis of tumor–immune–EMT model predictions via patient survival data from TCGA

We obtained primary tumor bulk mRNA sequencing and censored survival data for individuals monitored by cancer type from the TCGA^88^, accessed through the Genomic Data Commons portal^89^. We developed methods to study: (i) how the synergistic effects of EMT + inflammation compare to the effects of each of these individually; and (ii) the importance of mesenchymal proliferation rates in determining cancer prognosis (Supplementary Fig. S5), which allow us to test predictions from the agent-based model.

Given our focus on tumor invasiveness, we identified the TCGA outcome “disease-free interval” as the most relevant endpoint in our pathway analysis pipeline. However, in order to narrow the search over tumor types, in step (i) we use the “OS” endpoint. This choice is made since we use a proportional hazards model: the core assumption of this model is violated more frequently for DFI data than for OS data, due to challenges regarding clinical data curation. As Jatoi et. al note^90^, the proportionality of hazards with regard to DFI is violated in 27% of trials examined for BRCA, with regard to OS, it is violated in only 11% of trials.

Thus, for step (i), we use OS data to identify cases where synergistic effects due to the combination of EMT and inflammation pathways have a greater influence on survival than individual effects. For each cancer type, we obtained from MSigDB^91^ gene sets that contain EMT or Inflammation-related genes and, for each gene set, we tested whether EMT, inflammation, or the combination of these two effects best predicts OS. We selected for those gene sets that exhibit strong synergistic effects as identified by a Cox proportional-hazard (CPH) model. For tumor types where synergistic effects were most evident, we asked whether unsupervised clustering of patients, based on a low-dimensional representation of the combined gene set, could predict statistically significant differences in overall survival via the KM model. For tumor types where both the CPH and KM analysis were consistent, we conducted further analysis (ii) of the role played by proliferation on tumor invasiveness.

For step (ii), we use DFI data, as it best resembles the invasion-free survival metric used in modeling (in many cases, the disease may be undetected until it becomes invasive). We noticed that these times were bimodally distributed, suggesting that the gene regulation controlling invasion could be learned via binary classification. We determined the patient DFI class by fitting these invasion times to a two-component Gaussian mixture model, which assigns each patient to either high-DFI or low-DFI. We then used Gaussian process classification to learn the regulatory structure of a group of mesenchymal proliferation genes based on their ability to predict DFI class. Specifically, we clustered the genes based on the rank order statistics of their respective maximum-a-posteriori factor-analysis distances. Finally, we used simulations of the learned model to further examine the co-regulation of these genes, highlighting the interaction between immunity, tumor progression, and invasiveness in the context of treatment-response. Full details of the methods used for this analysis can be found in the Supplementary Text.

### Statistics and reproducibility

The multiscale model was developed in MATLAB (tested on version R2019b) and the analysis of TCGA data was performed in R (v4.0.1) using public data accessed from R using TCGAbiolinks and analyzed using packages (mclust,dbscan) and custom scripts, all of which are available online. Gaussian Process modeling with TCGA data was performed in MATLAB (R2020a) using the package GPstuff^92^. In each in silico simulation experiment, 1000 independent runs were performed. For survival analysis, KM and Cox proportional hazards tests were used to compare patient cohorts. A *p* value of < 0.05 was considered statistically significant.

### Reporting summary

Further information on research design is available in the Nature Research Reporting Summary linked to this article.

## Supplementary information

Supplementary Information

Reporting Summary

## Data Availability

The code required to specify and simulate the model used in this study is available at: https://github.com/drbergman/tumor-immune-emt-code. This includes the necessary code to generate all of the data that comprise the main figures associated with the paper. Data from the TCGA were downloaded from the GDC data portal: https://portal.gdc.cancer.gov/.

## Code availability

All software associated with model development, simulation, and analysis of TCGA data are available under an MIT license, on GitHub at: https://github.com/drbergman/tumor-immune-emt-code, and as a Zenodo archive: https://zenodo.org/record/4895005.
