# Supplementary material for: Modeling the effects of EMT-immune dynamics on carcinoma disease progression
Source: Commun Biol. 2021 Aug 18;4:983. doi: 10.1038/s42003-021-02499-y (PMC8373868; doi:10.1038/s42003-021-02499-y)
Supplement: Supplementary file 1 — Supplementary Information [file 42003_2021_2499_MOESM1_ESM.pdf]

Supplementary Material for Modeling the effects of EMT-immune dynamics on carcinoma disease progression

Daniel R. Bergman[1,†], Matthew K. Karikomi[1,†], Min Yu[2], Qing Nie[1,3,*], and Adam L. MacLean[1,4,*]

[1]Department of Mathematics, University of California, Irvine, Irvine, CA 92697
[2]Department of Stem Cell & Regenerative Medicine, Keck School of Medicine, University of Southern California, Los Angeles, CA 90033
[3]Department of Cell and Developmental Biology, University of California, Irvine, Irvine, CA 92697
[4]Department of Biological Sciences, University of Southern California, Los Angeles, CA 90089
[†]These authors contributed equally
[*]Correspondence: qnie@uci.edu (Q.N.); macleana@usc.edu (A.L.M.).

Contents

1 Supplementary Methods: Details of parameter values 3

2 Supplementary Methods: Multiscale model development 6
- 2.1 Tissue cell fate 6
  - 2.1.1 Proliferation 6
  - 2.1.2 Apoptosis 6
  - 2.1.3 Immune Clearance 7
  - 2.1.4 Rest in G_0 7
  - 2.1.5 Updating the immune system and EMT dynamics . . 8

3 Supplementary Methods: Analysis of data from TCGA 10
- 3.1 Overall Survival 10
  - 3.1.1 Cox-PH Model: Synergistic Pathway Identification . . 11
  - 3.1.2 KM Model: Synergistic Pathway Confirmation 11
- 3.2 Disease-Free Interval 12

3.2.1 DFI is a Clinical Analogue of Tumor Time to Invasion 12
3.2.2 Ontology-Based Investigation of Proliferation Pathways 12
3.2.3 Binary Classification of DFI Endpoints 13

4 Supplementary Tables: Cox-PH tables 15
4.1 BLCA 15
4.2 CESC 15
4.3 COAD 15
4.4 ESCA 16
4.5 KIRP 16
4.6 LIHC 17
4.7 PAAD 18
4.8 UCEC 19

5 Supplementary Tables: KM tables 21

6 Supplementary Figures 23

1 Supplementary Methods: Details of parameter values

Name	Description	Value (INFL high)	Units	Source
p	Rate of proliferation for tissue cells	0.28	Cycle^{-1}	[15]
d_C	Rate of apoptosis for tissue cells	0.14	Cycle^{-1}	[15]
ζ	Boolean for cell fate: mesenchymal (1) or epithelial (0)	$\{0, 1\}$	NA	NA
Δ_{MIE}	Mesenchymal immune evasion (MIE)	Varies	NA	This work
Δ_{MGA}	Mesenchymal growth arrest (MGA)	Varies	NA	This work
δ_A	Boolean for cell with (1) or without (0) apoptosis mutation	$\{0, 1\}$	NA	NA
Δ_A	Fractional decrease in rate of apoptosis following mutation	0.3	NA	Estimated
δ_{IE}	Boolean for cell with (1) or without (0) immune evasion mutation	$\{0, 1\}$	NA	NA
Δ_{IE}	Fractional increase in rate of immune evasion following mutation	0.48	NA	Estimated
δ_P	Boolean for cell with (1) or without (0) proliferation mutation	$\{0, 1\}$	NA	NA
Δ_P	Fractional increase in rate of proliferation following mutation	0.36	NA	Estimated
K_0	EC50 term for negative feedback of tissue cells on own proliferation	80	Cells	Estimated
K_1	EC50 term for probability of NK cell finding mutant cell	8	Cells	Estimated from [29]

K_2	EC50 term for Treg inhibition of cytotoxic functions	5 (0.025)	Cells / Volume	Simulated*
K_3	EC50 term for cumulative absorption of TGF-β	200	Amount / Volume	Simulated*
K_4	EC50 term for TGF-β activation of Tregs	50	Amount / Volume	Simulated*
E_{NK}	Rate of NKs clearing mutants	10 (30)	Cycle^{-1}	[29]
E_{CTL}	Rate of CTLs clearing mutants	200 (600)	Cycle^{-1}	[29]
σ_{NK}	NK source rate	1.3	Cells / Cycle	[29]
σ_{CTL}	CTL source rate per cleared mutant cell	100	Cells / (Mutants $\times$ Cycles)	[32]
σ_{Treg}	Treg source rate per cleared mutant cell	200	Cells / (Mutants $\times$ [TGF-β] $\times$ Cycles)	[32]
d_{NK}	NK death rate	0.13	Cycle^{-1}	[29]
d_{CTL}	CTL death rate	0.0260	Cycle^{-1}	[32]
d_{Treg}	Treg death rate	0.0260	Cycle^{-1}	[32]
k_{EMT}	Rate of EMT	0.01	[TGF-β]$^{-1}$	Simulated*
k_{MET}	Rate of MET	0.02	[TGF-β]$^{-1}$	Simulated*
σ	Standard deviation of noise in [TGF-β]	6	[TGF-β]	Estimated
τ_{max}	Max amount of TGF-β a cell can receive	500	[TGF-β]	Estimated
τ_{MUT}	TGF-β production rate by mutant cells	0.05	[TGF-β] / Cell / Cycle	Estimated
τ_{Treg}	TGF-β production rate by Tregs	0.5	[TGF-β] / Cell / Cycle	Estimated
Δt	Duration of cell cycle	18	Hours	[9]
–	Relative population threshold determining time to invasion	0.5	NA	Selected*
–	INFL High Duration	30	Cycles	Varied
–	INFL Low Duration	30	Cycles	Varied
T_{MES}	Mesenchymal threshold	0.7	NA	Simulated*

T_{EMT}	Threshold for undergoing EMT	2	NA	Simulated*
–	Max initial mutation probability after warmup	0.01	NA	[15]
–	Increase in probability of mutation per cell proliferation	0.0001	NA	[15]

Table S1: Description of all parameters used for simulation, along with the base values that define the low inflammatory state, and their units. Secondary value in parentheses is given if the parameter changes in the high inflammatory state. [TGF-β]: concentration of TGF-β. *Simulated denotes values that were identified following a parameter sweep. *Selected denotes parameters that were fixed by experimental design.

2 Supplementary Methods: Multiscale model development

2.1 Tissue cell fate

During each cell cycle, every cell randomly is assigned a cell fate from the following options:

- proliferation
- apoptosis
- immune clearance (by NKs or CTLs)
- rest in G_0

For each cell, the rates are normalized to probabilities which then are used to randomly determine what each cell does during the cell cycle.

2.1.1 Proliferation

There are four factors that contribute to the rate at which a cell proliferates. The first is a base proliferation rate that all cells have, p. Second, if the cell has a mutation in the proliferation pathway ($\delta_P = 1$), then the rate for proliferation is proportionally increased by Δ_P. Third, if the cell is mesenchymal ($\zeta = 1$), then the rate for proliferation is proportionally decreased by Δ_{MGA}, which stands for mesenchymal growth arrest. This lost proliferation for mesenchymal cells will later be used to increase their chance of resting. Fourth, there is a negative feedback of the cells on their own proliferation which is quantified by a Hill factor as a function of the tissue cell population, N_C, with EC50 term K_0. In total, the rate for proliferation is given by

$$\rho_P = \underbrace{p}_{\substack{\text{base}\\ \text{proliferation}}} \underbrace{(1 + \delta_P \Delta_P)}_{\substack{\text{proliferation}\\ \text{pathway mutation}}} \underbrace{(1 - \zeta \Delta_{\text{MGA}})}_{\substack{\text{mesenchymal}\\ \text{growth arrest}}} \underbrace{\frac{1}{1 + N_C/K_0}}_{\substack{\text{resource}\\ \text{competition}}} \tag{1}$$

2.1.2 Apoptosis

There are two factors that contribute to a cell's rate for undergoing apoptosis. There is a basal apoptosis rate that all cells experience, d_C for death. Second, if the cell has a mutation in the apoptosis pathway ($\delta_A = 1$), then the rate for undergoing apoptosis is proportionally decreased by Δ_A. In total, the rate for apoptosis is given by

$$\rho_A = \underbrace{d_C}_{\substack{\text{base}\\ \text{apoptosis}}} \underbrace{(1 - \delta_A \Delta_A)}_{\substack{\text{apoptosis}\\ \text{pathway mutation}}} \tag{2}$$

2.1.3 Immune Clearance

For both NK clearance and CTL clearance, the rates are built with the same factors but have different parameter values for NK and CTLs. First of all, the cell needs to be malignant ($\delta_{\text{MUT}} = 1$). Second, there is a Hill factor that captures the probability of an immune cell finding and interacting with the given tissue cell with EC50 term K_1. Third, NKs and CTLs have their own efficacy parameters, E_{NK} and E_{CTL}, which can be understood as the rate of immune clearance given an immune cell has found the mutated cell. Fourth, there is a decreasing Hill factor based on the number of Treg cells present with EC50 term K_2. Finally, there are two factors that proportionally decrease the rate of immune clearance depending on if the cell has an immune evasion mutation ($\delta_{\text{IE}} = 1$) or if it is mesenchymal ($\zeta = 1$) with respective decreases Δ_{IE} and Δ_{MIE}. In total, the rate of NK clearance is given by

$$\rho_{\text{NK}} = \underbrace{\delta_{\text{MUT}}}_{\substack{\text{only target}\\ \text{invasive cells}}} \underbrace{\frac{N_{\text{NK}}}{N_C/K_1 + N_{\text{NK}}}}_{\substack{\text{probability of}\\ \text{encounter}}} \underbrace{\frac{\overbrace{E_{\text{NK}}}^{\substack{\text{probability}\\ \text{of clearance}}}}{1 + N_{\text{Treg}}/K_2}}_{\substack{\text{Treg inhibition}\\ \text{of clearance}}} \underbrace{(1 - \delta_{\text{IE}}\Delta_{\text{IE}})}_{\substack{\text{immune evasion}\\ \text{pathway mutation}}} \underbrace{(1 - \zeta\Delta_{\text{MIE}})}_{\substack{\text{mesenchymal}\\ \text{immune evasion}}} \tag{3}$$

A similar formula holds for CTLs with only the number of CTLs and their efficacy being different from the above equation:

$$\rho_{\text{CTL}} = \underbrace{\delta_{\text{MUT}}}_{\substack{\text{only target}\\ \text{invasive cells}}} \underbrace{\frac{N_{\text{CTL}}}{N_C/K_1 + N_{\text{CTL}}}}_{\substack{\text{probability of}\\ \text{encounter}}} \underbrace{\frac{\overbrace{E_{\text{CTL}}}^{\substack{\text{probability}\\ \text{of clearance}}}}{1 + N_{\text{Treg}}/K_2}}_{\substack{\text{Treg inhibition}\\ \text{of clearance}}} \underbrace{(1 - \delta_{\text{IE}}\Delta_{\text{IE}})}_{\substack{\text{immune evasion}\\ \text{pathway mutation}}} \underbrace{(1 - \zeta\Delta_{\text{MIE}})}_{\substack{\text{mesenchymal}\\ \text{immune evasion}}} \tag{4}$$

2.1.4 Rest in G_0

The rate associated with rest is taken as 1 except in the case of mesenchymal cells. Recall that mesenchymal cells had their proliferation rate decreased by $1 - \zeta\Delta_{\text{MGA}}$ (see Eq. 2.1). The biological assumption here is that mesenchymal cells instead of proliferating will instead rest, so this lost proliferation rate is added to the resting rate. Hence, the rate of rest is given by

$$\rho_R = \underbrace{1}_{\substack{\text{base}\\ \text{rest rate}}} + \underbrace{\zeta p(1 + \delta_P\Delta_P)\Delta_{\text{MGA}}\frac{1}{1 + N_C/K_0}}_{\text{loss of proliferation due to being mesenchymal}} \tag{5}$$

Again, the reason for adding that term is due to the understanding that overall mesenchymal cells proliferate less as individual cells rest longer in the G_0 phase.

2.1.5 Updating the immune system and EMT dynamics

After the cell fates are determined and the results reflected in the system, additional steps are completed before the system moves on to a new cell cycle. First, the NK and CTL populations are reduced by the number of mutated cells that have been cleared. This represents the fact that individual immune cells lose efficacy as they carry out their effector functions. Second, all proliferating cells have a cell-specific probability of undergoing a driver mutation in one of the three pathways. If a cell is selected to mutate, the pathway mutation is chosen with probability 1/3 and the mutation is applied to that cell. If the cell does not mutate, then its per-cell probability of mutation during subsequent cell cycles increases by a fixed amount.

Next the EMT state of each cell is updated. This is achieved through updating the EMT score for cell i at time step j, $S_{i,j}$. $S_{i,j}$ takes values between 0 (epithelial) and 1 (mesenchymal), and is given by:

$$S_{i,j+1} = \begin{cases} S_{i,j} \exp\left(k_{\text{MET}}(\tau_i - T_{\text{EMT}})\right) & \tau_i < T_{\text{EMT}} \\ 1 - (1 - S_{i,j}) \exp\left(k_{\text{EMT}}(T_{\text{EMT}} - \tau_i)\right) & \tau_i \geq T_{\text{EMT}}, \end{cases} \tag{6}$$

where τ_i is the total level of TGF-β in cell i (given below), and the parameters are given in Table S1. T_{EMT} is a threshold value that defines cell fate, i.e., if $\tau_i > T_{\text{EMT}}$, then the cell state moves towards mesenchymal, and the EMT score increases towards 1. Otherwise, the cell state moves towards epithelial, and the EMT score decreases towards 0. The updated EMT score ($S_{i,j+1}$) is then compared to a threshold value, T_{MES}, and the cell commits to a mesenchymal (epithelial) state if the score is above (below) this threshold.

A key determinant of EMT is the level of TGF-β, both cell exogenous and endogenous. The total amount of TGF-β in the TME available for exogenous signaling, τ, is given by:

$$\tau = \tau_{\text{MUT}} N_{\text{MUT}} + \tau_{\text{Treg}} N_{\text{Treg}}, \tag{7}$$

which depends on the total number of mutant cells, N_{MUT} and the Treg cell population N_{Treg}. From this, the level of TGF-β in each individual cell i, τ_i, is calculated by:

$$\tau_i = \frac{\tau_{\max}}{N_C} \frac{\tau/K_3}{1 + \tau/K_3} + S_{i,j} + X_i, \quad X_i \sim N(0, \sigma^2) \tag{8}$$

where the first term of the RHS represents the level of exogenous TGF-β, which is modeled by a Hill function controlled by the total amount of TGF-β

in the TME (Eqn. 7). The second term $S_{i,j}$ is the EMT score, defined in Eqn. 6, and used here to characterize the endogenous TGF-β level. The third and final term is a term to characterize the noise in the amount of TGF-β received by each cell, which is assumed to be Gaussian. NB in Eqn. 8 the subscript j for time step is included on the EMT score S since there is an explicit dependence of S on the previous time step in Eqn. 6. We do not include the subscript j for other terms due to readability, although we note that the variables τ_i, N_C, and X_i are also dependent on the time step j. The amount of TGF-β available in the next cell cycle (Eqn. 7) is updated by determining the new number of mutated cells, N_{MUT}, and the number of Treg cells, N_{Treg} at the end of the cell cycle.

Finally, the immune populations are updated. NK cells obey the following differential equation:

$$N'_{\text{NK}} = \sigma_{\text{NK}} - d_{\text{NK}} N_{\text{NK}} \tag{9}$$

which is discretized to

$$N_{\text{NK}}(k+1) = \left(N_{\text{NK}}(k) - \frac{\sigma_{\text{NK}}}{d_{\text{NK}}} \right) \exp(-d_{\text{NK}} \Delta t) + \frac{\sigma_{\text{NK}}}{d_{\text{NK}}} \tag{10}$$

CTL and Treg cells (adaptive immune components) are not activated until malignant cells are being cleared from the system. Treg recruitment is upregulated by TGF-β, which will be incorporated via a Hill function with EC50 term K_4. Let $N^*_{\text{MUT}}(k)$ represent the number of malignant cells cleared by the immune system during cell cycle k. Then the following differential equations govern the CTL and Treg population dynamics:

$$\begin{aligned} N'_{\text{CTL}} &= \sigma_{\text{CTL}} N^*_{\text{MUT}} - d_{\text{CTL}} N_{\text{CTL}} \\ N'_{\text{Treg}} &= \sigma_{\text{Treg}} N^*_{\text{MUT}} \frac{\tau}{1+\tau/K_4} - d_{\text{Treg}} N_{\text{Treg}} \end{aligned} \tag{11}$$

Discretized, these are:

$$\begin{aligned} N_{\text{CTL}}(k+1) &= (N_{\text{CTL}}(k) - \sigma_{\text{CTL}} N^*_{\text{MUT}}(k)/d_{\text{CTL}}) \exp(-d_{\text{CTL}} \Delta t) + \sigma_{\text{CTL}} N^*_{\text{MUT}}(k)/d_{\text{CTL}} \\ N_{\text{Treg}}(k+1) &= \left(N_{\text{Treg}}(k) - \frac{\sigma_{\text{Treg}} N^*_{\text{MUT}}(k)}{d_{\text{Treg}}} \frac{\tau(k)}{1+\tau(k)/K_4} \right) \exp(-d_{\text{Treg}} \Delta t) \\ &\quad + \frac{\sigma_{\text{Treg}} N^*_{\text{MUT}}(k)}{d_{\text{Treg}}} \frac{\tau(k)}{1+\tau(k)/K_4} \end{aligned} \tag{12}$$

3 Supplementary Methods: Analysis of data from TCGA

The Cancer Genome Atlas (TCGA) [7] provides multiple clinical endpoints, including overall survival (OS) and disease-free interval (DFI) [23]. In order to investigate the link between EMT, inflammation and invasive phenotype, we corroborate our model with clinical data from TCGA in a two pronged approach:

- **Overall Survival:** Test whether EMT and inflammation can jointly separate clinical cohorts based on the OS endpoint for a selection of cancer sub-types (Section 3.1).
- **Disease-Free Interval:** Identify pathway genes which regulate the proliferation/tumor-invasiveness axis in the context of a synergistic EMT/Inflam effect (Section 3.2).

Our analysis of tumor invasiveness due to mesenchymal growth arrest, in the context of EMT and inflammation takes place in two steps (fig S5.A-D): First, identify relevant cancers by A) defining sets of genes which represent some union of inflammation and EMT pathways while simultaneously having a quantitatively greater effect on overall survival than the component pathways acting alone, and B) simulating the dosage effect of proliferation markers on tumor invasiveness, for cancer types where a synergistic EMT/Inflam pathway was identified in (A). Following guidelines published in [23], we investigated the 14 TCGA tumor types recommended for both OS and DFI analysis.

3.1 Overall Survival

While immunological interactions and EMT are known to be related [14], there is uncertainty regarding both the individual pathways which govern this dependence and the extent to which the interaction between inflammation and EMT is synergistic. Our approach identifies pathways by gene set (among all pairwise combinations of EMT ($\mathcal{GS}_{\text{EMT}}$) and inflammation ($\mathcal{GS}_{\text{Inflam}}$) gene sets available from MSigDB [21] for which the synergistic relationship between EMT and inflammation has a greater effect on OS than either process individually. Because only a subset of the 14 TCGA tumor-types we analyzed contains at least one such pathway, this approach results in a search over tumor types whose biology is governed by the EMT/inflammation/ interaction. Thus, OS analysis takes place in the following steps, which we detail in the remainder of 3.1:

1. (3.1.1) For each tumor-type, check Cox model for $\{(x, y)|x \in \mathcal{GS}_{\text{EMT}} \wedge y \in \mathcal{GS}_{\text{Inflam}}\}$

2. (3.1.2) For each (x, y), check KM model for unsupervised DBSCAN clusters

3.1.1 Cox-PH Model: Synergistic Pathway Identification

For each combination of gene sets, we created three (one-dimensional) UMAP projections [25] of the data, one each from A) the EMT genes, B) the Inflammation genes, and C) the concatenation of (A) and (B). This yielded a three-dimensional projection of the data, on which we build a Cox proportional hazard model (CoxPH). We identified several gene set combinations (combos) for which the global statistical significance (by likelihood ratio test) of the corresponding model was high ($p_{LR} \leq 5e-2$), as were all three predictors, but for which the hazard ratios for the concatenation embedding were at least 5% greater in magnitude than either EMT or inflammation alone.

Prior to CoxPH analysis, the proportional hazard assumption was tested and only tumor-type/combos were retained whose Schoenfeld residual was equal to 0 [17]. Our screen identified 13 tumor-type/combos across 8 tumor types: urothelial bladder carcinoma (BLCA, table S2), Cervical Squamous Cell Carcinoma and Endocervical Adenocarcinoma (CESC, table S3), colon adenocarcinoma (COAD, S4), esophageal carcinoma (ESCA, S5), cervical kidney renal papillary cell carcinoma (KIRP, tables S6, S7), liver hepatocellular carcinoma (LIHC, tables S8,S9), pancreatic adenocarcinoma (PAAD, tables S10,S11), and uterine corpus endometrial carcinoma (UCEC, tables S12,S13,S14). See supplementary file "OS_test_results.csv" for full results (all tumor-type/combos).

3.1.2 KM Model: Synergistic Pathway Confirmation

In order to provide further confirmation of a relationship between survival in these four cancers and the synergistic activation of relevant pathways, we tested the separation (adjusted $p_{\text{log rank}} \leq 0.05$, [17]) of KM models fitted to subgroups defined unsupervised hierarchical density-based clustering [5, 12] (DBSCAN) of the UMAP-embedded combined gene set. We guided the unsupervised clustering by scaling down the minimum neighborhood size (starting with 30 patients) until the number of clusters was at least two. In addition to assigning cluster labels, DBSCAN determines outliers based on the the neighborhood structure of the graph [5,12]. In our KM models and in subsequent analysis of DFI prediction, these outliers were discarded in order to ensure that groups of patients were maximally homogeneous with respect to EMT/inflammation. A single gene set combo met these criteria for the following cancers: BLCA (table S15, survival plot fig.S6), LIHC (table S16, survival plot fig.S7), and UCEC (table S17, survival plot fig.S8).

This analysis robustly identified BLCA, LIHC, and UCEC as cancers for which synergistic interaction between EMT and inflammation is the primary

driver of patient survival. Our approach has several advantages. First, we utilized the MSigDB resource [21] in order to optimize the search space over relevant pathways. This allows the large volume of prior knowledge encoded in this database to guide exploratory data analysis that would otherwise be impossible or impractical at the transcriptomic scale [37]. Second, our use of dimensionality-reduction provides the following two-fold advantage: clear interpretation of the synergistic response between EMT and INFLAM and the compression of the parameter space to only three predictors, which means that sensitive prediction of survival can be carried out on the limited number of primary tumor samples in our data set. In the sequel, we address the role of mesenchymal proliferation pathways in the invasiveness of these tumors, by utilizing the disease-free interval (DFI) endpoint [23], rather than the OS endpoint.

3.2 Disease-Free Interval

3.2.1 DFI is a Clinical Analogue of Tumor Time to Invasion

Our agent-based simulations cover the incremental progression from in-situ to invasive disease from a homogeneous initial point, whereas the data in TCGA address how cancer may progress following treatment, thus comparisons between model and data should be made carefully. Nonetheless recent clinical and experimental evidence suggests that core cellular tumor dynamics are at play both during the tumor progression addressed by the model, and post-treatment progression described in data from TCGA. Of particular note, the plasticity of tumor cells allows them to evade treatment by undergoing post-treatment processes resembling the de-novo appearance of cancer [33].

3.2.2 Ontology-Based Investigation of Proliferation Pathways

As stated above, we predict that for certain EMT and inflammatory environments, the time to invasion is maximized by a specific proliferative regime, where the proliferative potential of a transformed tumor cell is being held in check by mesenchymal growth arrest programs. Therefore we investigate the timing of invasion as a function of proliferation by searching for proliferative regimes where the Disease Free Interval (DFI) is maximized for patients in remission after treatment. In contrast to the search-based strategy above, we used the Gene Ontology (GO) resource [1, 8] to select an appropriate pathway for this analysis. GO is designed to provide a semantic index of genes, allowing gene lists to be retrieved interactively by simply browsing its hierarchy. We selected GO:0010463 (Mesenchymal Cell Proliferation) for our analysis of proliferation-dependent DFI.

3.2.3 Binary Classification of DFI Endpoints

Binarization of survival endpoints has previously been explored [3, 6, 10, 16, 18, 20, 22]. In contrast to previous approaches which utilize a pre-determined time threshold for the response (e.g. early and late relapse), we utilized an imputed high/low risk classification scheme based on a two-component Gaussian mixture model, which implicitly deals with cancer-specific thresholds. This approach was motivated by the observation that in all three cases, the DFI exibited multiple modes with cancer-specific thresholds: BLCA (fig.S9), LIHC (fig.S11), UCEC (fig.S10). Under this scheme, a tumor with a short DFI represents highly invasive disease for which the time-to-invasion is short.

Summary of the Model

The log counts from TCGA bulk mRNA sequencing for 52 genes are used to predict the computed DFI-class for each patient. The list of genes includes the 42 human genes from GO:0010463 "mesenchymal cell proliferation" (which omits all but LRP5 among known receptors for WNT2/11/5A) augmented with missing receptors for those Wnts: FZD2, FZD4, FZD6, FZD8, ROR1, ROR2, RYK, and LRP6. The model encodes the response y as either $+1$ or -1 (Eq. 13) for high-DFI and low-DFI respectively and is fitted via a generalized form of Bayesian logistic regression [30] using the expression levels of these genes as predictors. Since the likelihood (Eq. 13) is non-Gaussian, the posterior (Eq. 15) becomes analytically intractible, so expectation propagation (EP) is used to approximate it during inference and hyperparameter optimization [30]. Inference and hyperparameter optimization were performed using the gpstuff toolbox for MATLAB [34].

Our model simultaneously considers the (gene-expression of) multiple distinct biological pathways using the product of squared-exponential kernels over the predictor genes (Eq. 16). Within the context of the GP classifier, this kernel specifies the covariance of the joint distribution over any subset of the input data. The constant term σ_0, magnitude σ^2, and gene-wise length scale λ_d are given priors (Eq. 17, 18, 19, 20) which facilitate the discovery of their MAP values by EP. This basic structure assumes little prior knowledge

about the predictors, while offering good out of sample prediction accuracy.

$$p(\boldsymbol{y}|\boldsymbol{f}) = \prod_{i=1}^{n} \frac{1}{1 + exp(y_i f_i)} \quad (13)$$

$$p(\boldsymbol{f}|\boldsymbol{X}, \theta) = \mathcal{N}(\boldsymbol{f}|\boldsymbol{0}, \boldsymbol{K}) \quad (14)$$

$$p(\boldsymbol{f}|\boldsymbol{X}, \boldsymbol{y}, \theta) = \frac{p(\boldsymbol{f}|\boldsymbol{X}, \theta)}{p(\boldsymbol{X}|\theta)} \prod_{i=1}^{n} p(y_i|f_i) \quad (15)$$

$$\boldsymbol{K}(\boldsymbol{x}, \boldsymbol{x'}) = \sigma_0 + \sigma^2 exp \left[-\frac{1}{2} \sum_{d=1}^{D} \left(\frac{\boldsymbol{x} - \boldsymbol{x'}}{\lambda_d} \right)^2 \right] \quad (16)$$

$\boldsymbol{x}$ and $\boldsymbol{x'}$ any two patients

$$log(\sigma_0) \sim \mathcal{N}(0, 0.1) \quad (17)$$

$$log(\sigma^2) \sim \mathcal{N}(1, 0.25) \quad (18)$$

$$log(\lambda_d) \sim \mathcal{N}(1, \Sigma_0) \quad (19)$$

$$\Sigma_0 \sim \mathcal{IG}(3, 1) \quad (20)$$

This model achieves very high (~ 1) LOO-CV accuracy on the training data, so it is instructive to measure its performance relative to linear SVM on the sub-cohorts (noisy resamplings of the patient data) used for clustering. the average classification performance over all 1000 subcohorts is shown in the following table:

Type	$-\log(p(y))$	Naive	Linear SVM	GP
LIHC	197.800	0.582	0.582	1.0
BLCA	106.053	0.686	0.689	1.0
UCEC	62.321	0.708	0.726	1.0

Above, we list the leave-one-out cross-validated (LOO-CV) classification accuracy in each case for GP, and the 5-fold cross-validation accuracy for linear SVM, computed using the MATLAB Optimization Toolbox. The LOO-CV approach of [28] utilizing the cavity distribution of the EP likelihood approximation is utilized for tractability. This approach aims to discover the out of sample prediction accuracy for the model while simultaneously using all the data [35]. Compared to linear SVM [13], the GP classifier for all cancers achieves 100% LOO-CV, while SVM achieves only a modest improvement over naive (selecting high-DFI for all patients) for BLCA and UCEC, while failing improve the naive estimate for LIHC. This latter result is consistent with the higher negative log marginal likelihood ($-\log(p(y))$) for LIHC, indicating that the association between our chosen markers and the DFI endpoint is less justified. Therefore, LIHC was excluded from further analysis.

4 Supplementary Tables: Cox-PH tables

4.1 BLCA

	Dependent variable:
	time
EMT	-0.045^{**}
	(0.018)
INFLAM	0.025^{***}
	(0.009)
BOTH	0.082^{***}
	(0.021)
Observations	401
R^2	0.056
Max. Possible R^2	0.991
Log Likelihood	−924.075
Wald Test	22.290^{***} (df = 3)
LR Test	23.300^{***} (df = 3)
Score (Logrank) Test	22.401^{***} (df = 3)
Note:	*p<0.1; **p<0.05; ***p<0.01

Table S2: Gotzman EMT vs. GO Pos Acute Inflam Ant
GOTZMANN_EPITHELIAL_TO_MESENCHYMAL_TRANSITION_UP
vs.
GO_POSITIVE_REGULATION_OF_ACUTE_INFLAMMATORY_RESPONSE_TO_ANTIGENIC_STIMULUS

4.2 CESC

	Dependent variable:
	time
EMT	-0.100^{***}
	(0.032)
INFLAM	-0.067^{***}
	(0.023)
BOTH	0.092^{**}
	(0.041)
Observations	291
R^2	0.060
Max. Possible R^2	0.910
Log Likelihood	−340.512
Wald Test	18.320^{***} (df = 3)
LR Test	18.083^{***} (df = 3)
Score (Logrank) Test	18.630^{***} (df = 3)
Note:	*p<0.1; **p<0.05; ***p<0.01

Table S3: GO Pos EMT vs. GO Leuk Act
GO_POSITIVE_REGULATION_OF_EPITHELIAL_TO_MESENCHYMAL_TRANSITION
vs.
GO_LEUKOCYTE_ACTIVATION_INVOLVED_IN_INFLAMMATORY_RESPONSE

4.3 COAD

	Dependent variable:
	time
EMT	-0.142^{***}
	(0.041)
INFLAM	0.067^{**}
	(0.026)
BOTH	0.126^{***}
	(0.048)
Observations	276
R^2	0.048
Max. Possible R^2	0.904
Log Likelihood	-316.811
Wald Test	15.000^{***} (df = 3)
LR Test	13.455^{***} (df = 3)
Score (Logrank) Test	14.372^{***} (df = 3)
Note:	*p<0.1; **p<0.05; ***p<0.01

Table S4: Hollern EMT Breast vs. GO Leuk Act
HOLLERN_EMT_BREAST_TUMOR_UP
vs.
GO_LEUKOCYTE_ACTIVATION_INVOLVED_IN_INFLAMMATORY_RESPONSE

4.4 ESCA

	Dependent variable:
	time
EMT	0.086^{**}
	(0.038)
INFLAM	-0.113^{***}
	(0.042)
BOTH	0.185^{***}
	(0.064)
Observations	183
R^2	0.052
Max. Possible R^2	0.972
Log Likelihood	-321.566
Wald Test	9.760^{**} (df = 3)
LR Test	9.838^{**} (df = 3)
Score (Logrank) Test	9.802^{**} (df = 3)
Note:	*p<0.1; **p<0.05; ***p<0.01

Table S5: GO Cardiac EMT vs. GO Neg Acute Inf

GO_CARDIAC_EPITHELIAL_TO_MESENCHYMAL_TRANSITION
vs.
GO_NEGATIVE_REGULATION_OF_ACUTE_INFLAMMATORY_RESPONSE

4.5 KIRP

	Dependent variable:
	time
EMT	-0.090^{**}
	(0.041)
INFLAM	-0.040^{***}
	(0.015)
BOTH	0.103^{**}
	(0.040)
Observations	287
R^2	0.101
Max. Possible R^2	0.769
Log Likelihood	−194.964
Wald Test	35.480^{***} (df = 3)
LR Test	30.644^{***} (df = 3)
Score (Logrank) Test	41.090^{***} (df = 3)
Note:	*p<0.1; **p<0.05; ***p<0.01

Table S6: GO Reg EMT vs. GO Neg Acute Inf

GO_REGULATION_OF_EPITHELIAL_TO_MESENCHYMAL_TRANSITION
vs.
GO_NEGATIVE_REGULATION_OF_ACUTE_INFLAMMATORY_RESPONSE

	Dependent variable:
	time
EMT	-0.129^{***}
	(0.033)
INFLAM	-0.068^{***}
	(0.026)
BOTH	0.121^{***}
	(0.030)
Observations	287
R^2	0.113
Max. Possible R^2	0.769
Log Likelihood	−193.046
Wald Test	44.270^{***} (df = 3)
LR Test	34.480^{***} (df = 3)
Score (Logrank) Test	52.588^{***} (df = 3)
Note:	*p<0.1; **p<0.05; ***p<0.01

Table S7: GO Reg EMT vs. GO Neg Inf

GO_REGULATION_OF_EPITHELIAL_TO_MESENCHYMAL_TRANSITION
vs.
GO_NEGATIVE_REGULATION_OF_INFLAMMATORY_RESPONSE

4.6 LIHC

	Dependent variable:
	time
EMT	-0.029^{**}
	(0.012)
INFLAM	-0.159^{**}
	(0.065)
BOTH	0.181^{***}
	(0.062)
Observations	364
R^2	0.058
Max. Possible R^2	0.974
Log Likelihood	−654.198
Wald Test	20.970^{***} (df = 3)
LR Test	21.748^{***} (df = 3)
Score (Logrank) Test	21.321^{***} (df = 3)
Note:	*p<0.1; **p<0.05; ***p<0.01

Table S8: GO Cardiac EMT vs. Zhou Inf FIMA Up

GO_REGULATION_OF_CARDIAC_EPITHELIAL_TO_MESENCHYMAL_TRANSITION
vs.
ZHOU_INFLAMMATORY_RESPONSE_FIMA_UP

	Dependent variable:
	time
EMT	-0.026^{**}
	(0.012)
INFLAM	-0.027^{***}
	(0.010)
BOTH	0.033^{**}
	(0.017)
Observations	364
R^2	0.046
Max. Possible R^2	0.974
Log Likelihood	−656.570
Wald Test	16.730^{***} (df = 3)
LR Test	17.005^{***} (df = 3)
Score (Logrank) Test	17.070^{***} (df = 3)
Note:	*p<0.1; **p<0.05; ***p<0.01

Table S9: GO Reg EMT Endo vs. GO Mac Inf Prot 1 Alpha

GO_REGULATION_OF_EPITHELIAL_TO_MESENCHYMAL_TRANSITION_INVOLVED_IN_ENDOCARDIAL_CUSHION_FORMATION
vs.
GO_MACROPHAGE_INFLAMMATORY_PROTEIN_1_ALPHA_PRODUCTION

4.7 PAAD

	Dependent variable:
	time
EMT	-0.036^{**}
	(0.018)
INFLAM	-0.063^{**}
	(0.029)
BOTH	0.052^{***}
	(0.018)
Observations	177
R^2	0.061
Max. Possible R^2	0.991
Log Likelihood	-407.162
Wald Test	10.610^{**} (df = 3)
LR Test	11.187^{**} (df = 3)
Score (Logrank) Test	10.742^{**} (df = 3)
Note:	*p<0.1; **p<0.05; ***p<0.01

Table S10: Alonso Met EMT Down vs. GO Neg Reg Inf
ALONSO_METASTASIS_EMT_DN
vs.
GO_NEGATIVE_REGULATION_OF_INFLAMMATORY_RESPONSE

	Dependent variable:
	time
EMT	-0.099^{***}
	(0.033)
INFLAM	-0.043^{**}
	(0.022)
BOTH	0.070^{**}
	(0.029)
Observations	177
R^2	0.099
Max. Possible R^2	0.991
Log Likelihood	-403.487
Wald Test	17.620^{***} (df = 3)
LR Test	18.536^{***} (df = 3)
Score (Logrank) Test	17.776^{***} (df = 3)
Note:	*p<0.1; **p<0.05; ***p<0.01

Table S11: Jechlinger EMT Down vs. GO Pos Reg Cyto Prod
JECHLINGER_EPITHELIAL_TO_MESENCHYMAL_TRANSITION_DN
vs.
GO_POSITIVE_REGULATION_OF_CYTOKINE_PRODUCTION_INVOLVED_IN_INFLAMMATORY_RESPONSE

4.8 UCEC

	Dependent variable:
	time
EMT	-0.158^{***}
	(0.045)
INFLAM	-0.140^{**}
	(0.062)
BOTH	0.216^{***}
	(0.075)
Observations	174
R^2	0.081
Max. Possible R^2	0.789
Log Likelihood	−127.984
Wald Test	14.660^{***} (df = 3)
LR Test	14.652^{***} (df = 3)
Score (Logrank) Test	14.971^{***} (df = 3)
Note:	*p<0.1; **p<0.05; ***p<0.01

Table S12: Alonso Met EMT Up vs. Fulcher Inf Resp Lectin LPS Down

ALONSO_METASTASIS_EMT_UP
vs.
FULCHER_INFLAMMATORY_RESPONSE_LECTIN_VS_LPS_DN

	Dependent variable:
	time
EMT	-0.179^{***}
	(0.054)
INFLAM	-0.047^{**}
	(0.019)
BOTH	0.232^{***}
	(0.079)
Observations	174
R^2	0.088
Max. Possible R^2	0.789
Log Likelihood	−127.269
Wald Test	11.950^{***} (df = 3)
LR Test	16.082^{***} (df = 3)
Score (Logrank) Test	12.639^{***} (df = 3)
Note:	*p<0.1; **p<0.05; ***p<0.01

Table S13: GO Cardiac EMT vs. GO Reg Inf Resp Wound

GO_CARDIAC_EPITHELIAL_TO_MESENCHYMAL_TRANSITION
vs.
GO_REGULATION_OF_INFLAMMATORY_RESPONSE_TO_WOUNDING

	Dependent variable:
	time
EMT	-0.038^{**}
	(0.019)
INFLAM	-0.172^{***}
	(0.044)
BOTH	0.174^{***}
	(0.050)
Observations	174
R^2	0.094
Max. Possible R^2	0.789
Log Likelihood	-126.768
Wald Test	21.670^{***} (df = 3)
LR Test	17.084^{***} (df = 3)
Score (Logrank) Test	16.551^{***} (df = 3)
Note:	*p<0.1; **p<0.05; ***p<0.01

Table S14: GO Card EMT vs. Wunder Inf Resp Chol Up
GO_CARDIAC_EPITHELIAL_TO_MESENCHYMAL_TRANSITION
vs.
WUNDER_INFLAMMATORY_RESPONSE_AND_CHOLESTEROL_UP

5 Supplementary Tables: KM tables

	N	Observed	Expected	(O-E)^2/E	(O-E)^2/V
Cluster 1	118	35.00	54.28	6.85	11.61
Cluster 2	52	25.00	19.29	1.69	1.99
Cluster 3	38	20.00	15.50	1.31	1.49
Cluster 4	93	54.00	44.93	1.83	2.79

p=0.008

Table S15: Gotzman EMT vs. GO Pos Acute Inflam Ant
GOTZMANN_EPITHELIAL_TO_MESENCHYMAL_TRANSITION_UP
vs.
GO_POSITIVE_REGULATION_OF_ACUTE_INFLAMMATORY_RESPONSE_TO_ANTIGENIC_STIMULUS

k

	N	Observed	Expected	(O-E)^2/E	(O-E)^2/V
Cluster 1	160	69.00	49.64	7.55	15.43
Cluster 2	110	32.00	51.36	7.30	15.43

p=5e-8

Table S16: GO Reg EMT Endo vs. GO Mac Inf Prot 1 Alpha
GO_REGULATION_OF_EPITHELIAL_TO_MESENCHYMAL_TRANSITION_INVOLVED_IN_ENDOCARDIAL_CUSHION_FORMATION
vs.
GO_MACROPHAGE_INFLAMMATORY_PROTEIN_1_ALPHA_PRODUCTION

	N	Observed	Expected	(O-E)^2/E	(O-E)^2/V
Cluster 1	88	21.00	14.86	2.54	5.12
Cluster 2	76	9.00	15.14	2.49	5.12

p=0.02

Table S17: GO Cardiac EMT vs. GO Reg Inf Resp Wound
GO_CARDIAC_EPITHELIAL_TO_MESENCHYMAL_TRANSITION
vs.
GO_REGULATION_OF_INFLAMMATORY_RESPONSE_TO_WOUNDING

Gene(s)	Cancer	Effect	Eff.Type	Citation
Wnts	Both	Onc	NA	[2, 4, 19, 26, 27]
FGFR2	BLCA	Supp	NA	[31]
FGFR2	UCEC	Onc	NA	[11]
FBXW4	BLCA	Supp	NA	[24]
HAND2	BLCA	Supp	NA	[36]
HAND2	UCEC	Supp	NA	[11]
FOXF1	BLCA	Supp	NA	New
FGFR2+FBXW4	BLCA	Supp	Coop	New
FGFR2+HAND2	UCEC	Supp	Ant	New

Table S18: Summary of reported findings. Column Key: Genes = the genes in the specified relationship, Cancer = BLCA or UCEC, Effect = Oncogenic or Suppressor, Effect Type = NA (single gene), Antagonistic or Cooperative, Status = Known or Unknown, Citation = reference utilized in the manuscript to reference a known effect

6 Supplementary Figures

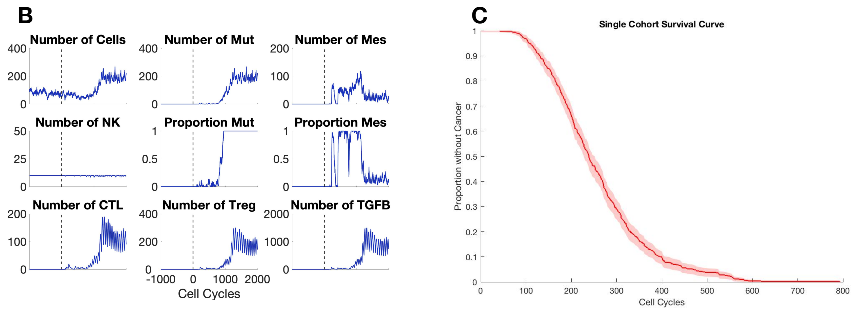

Figure S1: A. Single patient trajectory without immune cells targeting cells with pathways mutations. Compare to Fig. 1B. B. Sample cohort survival curve without immune cells targeting cells with pathways mutations. Compare to Fig. 1C.

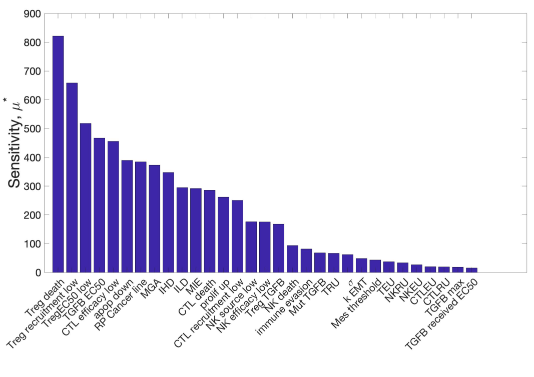

Figure S2: Morris-OAT global sensitivity without immune cells targeting cells with pathways mutations. Compare to Fig. 2.

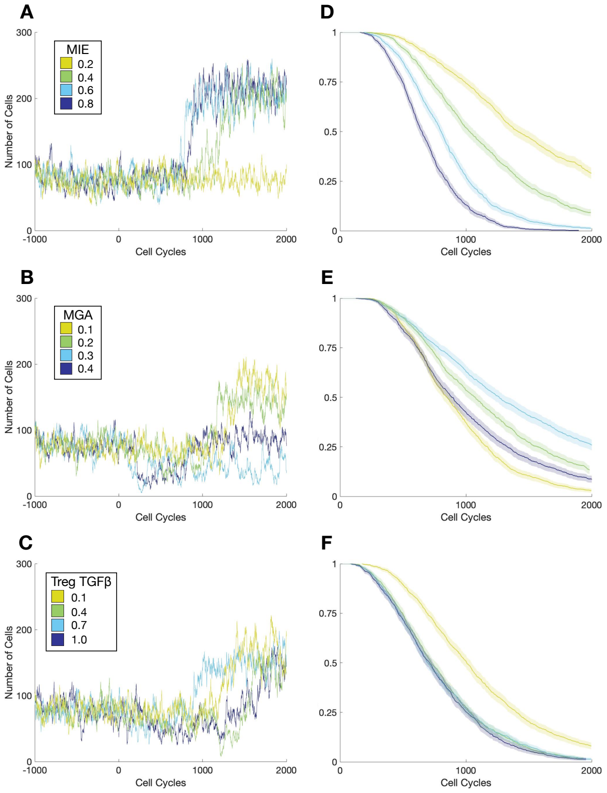

Figure S3: Effects of mesenchymal tumor cell properties on the Time to Invasion without immune cells targeting cells with pathways mutations. Compare to Fig. 3.

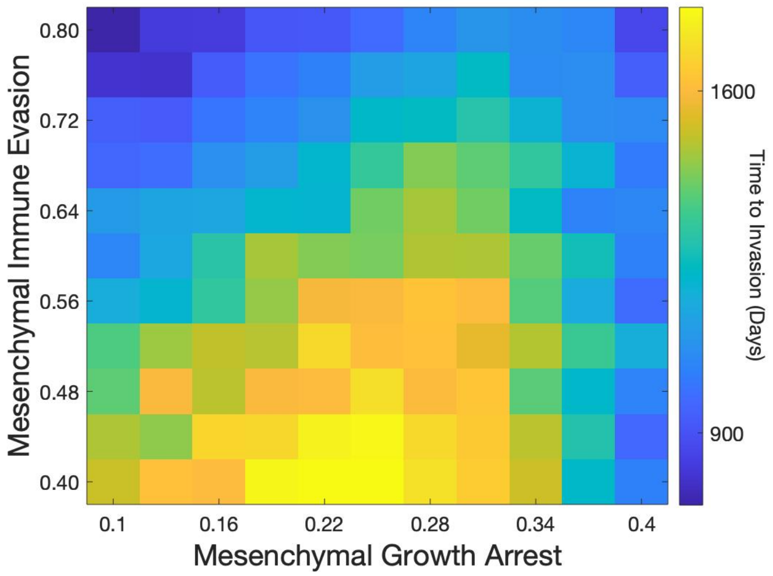

Figure S4: Summary of mesenchymal tumor cell properties on the Time to Invasion without immune cells targeting cells with pathways mutations. Compare to Fig. 4E.

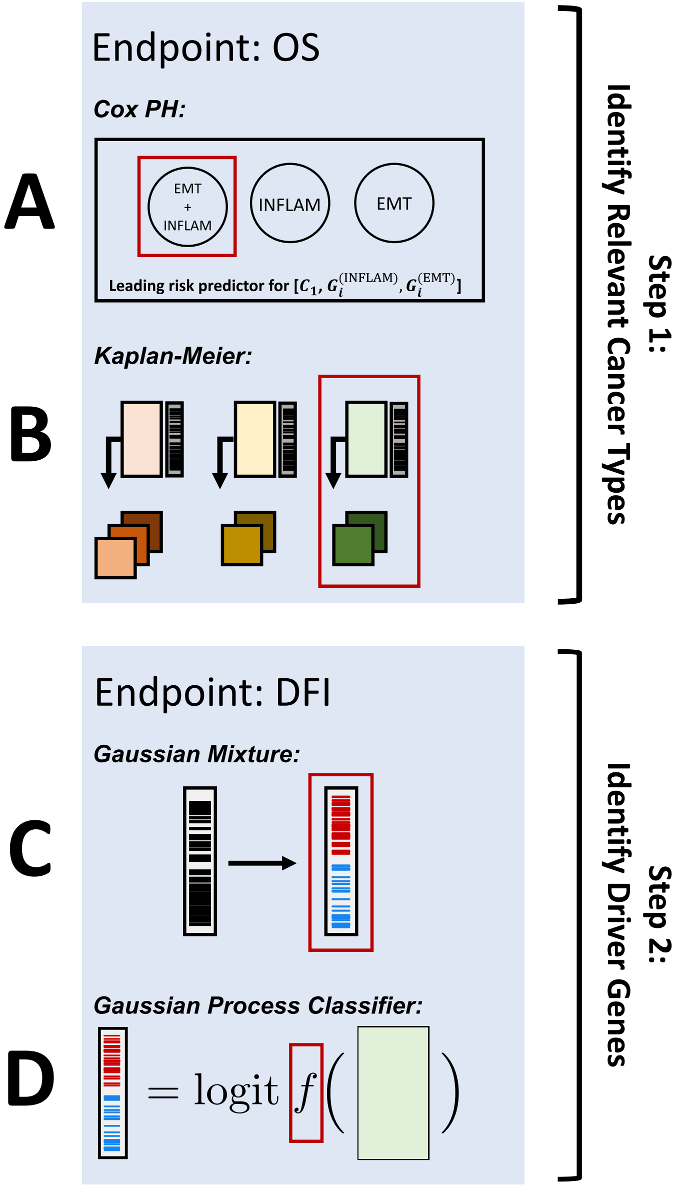

Figure S5: Schematic of overall survival (OS) and disease-free interval (DFI) analysis on TCGA data. A-B (Step 1): Find cancer types in which EMT and inflammation act synergistically on overall survival. C-D (Step 2): For cancer types identified in Step 1, identify proliferation pathways that regulate the invasiveness of these cancers. A) For each cancer type, identify pairs of inflammation and EMT gene sets where the UMAP projection over their union has a higher (magnitude) hazard ratio than either of its constituents in a three-predictor Cox-PH model with OS response in that cancer type. B) Identify cancer types, for which unsupervised DBSCAN clustering over the 1D UMAP projection of one or more EMT/Inflam union sets yields clusters whose KM survival curves are different. C) Impute the DFI-high/low class based on a two-component Gaussian mixture model of the published disease-free interval time in days. D) Identify relationships between proliferation pathways and tumor invasiveness using a GP classifier trained on the computed DFI-class from (C) and mRNA sequencing for the list of proliferation genes.

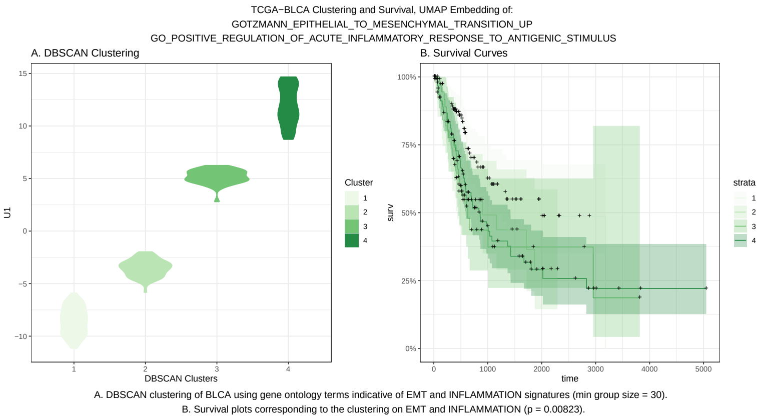

Figure S6: DBSCAN clusters for combined EMT+INFLAM embedding of BLCA patients and corresponding Kaplan-Meier (KM) model.

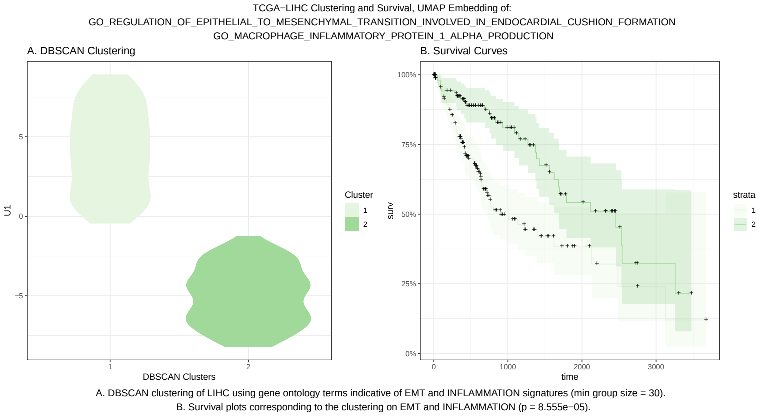

Figure S7: DBSCAN clusters for combined EMT+INFLAM embedding of LIHC patients and corresponding Kaplan-Meier (KM) model.

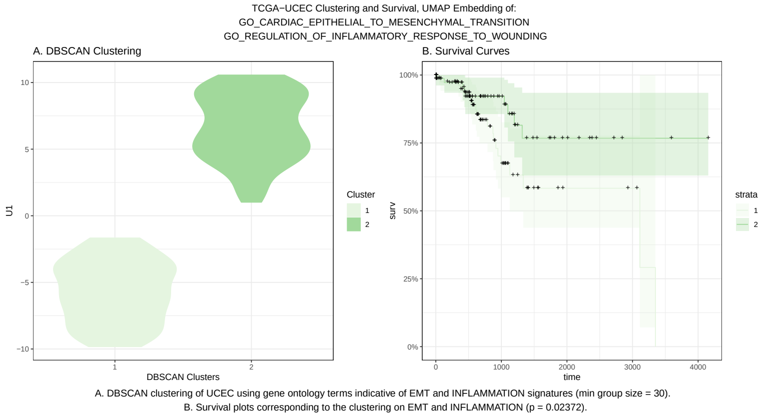

Figure S8: DBSCAN clusters for combined EMT+INFLAM embedding of UCEC patients and corresponding Kaplan-Meier (KM) model.

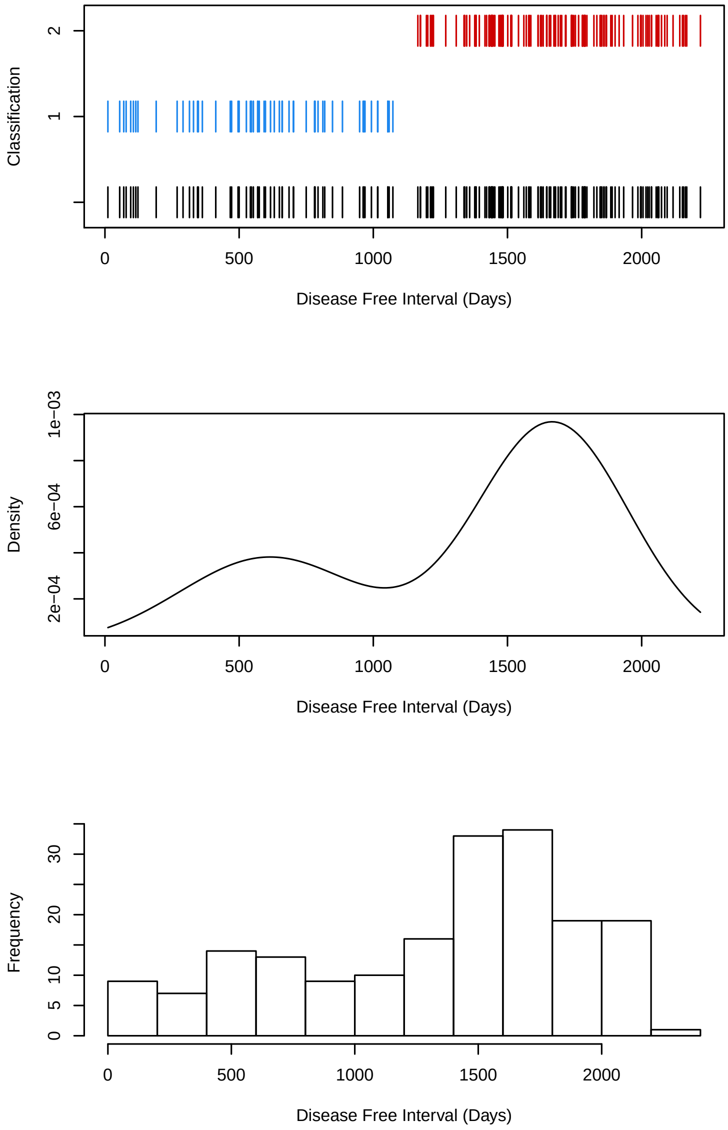

Figure S9: A. Classification of BLCA patients in short DFI (blue) and long DFI (red). B. 2-component Gaussian mixture density corresponding to the above classification. C. Histogram of DFI for patients.

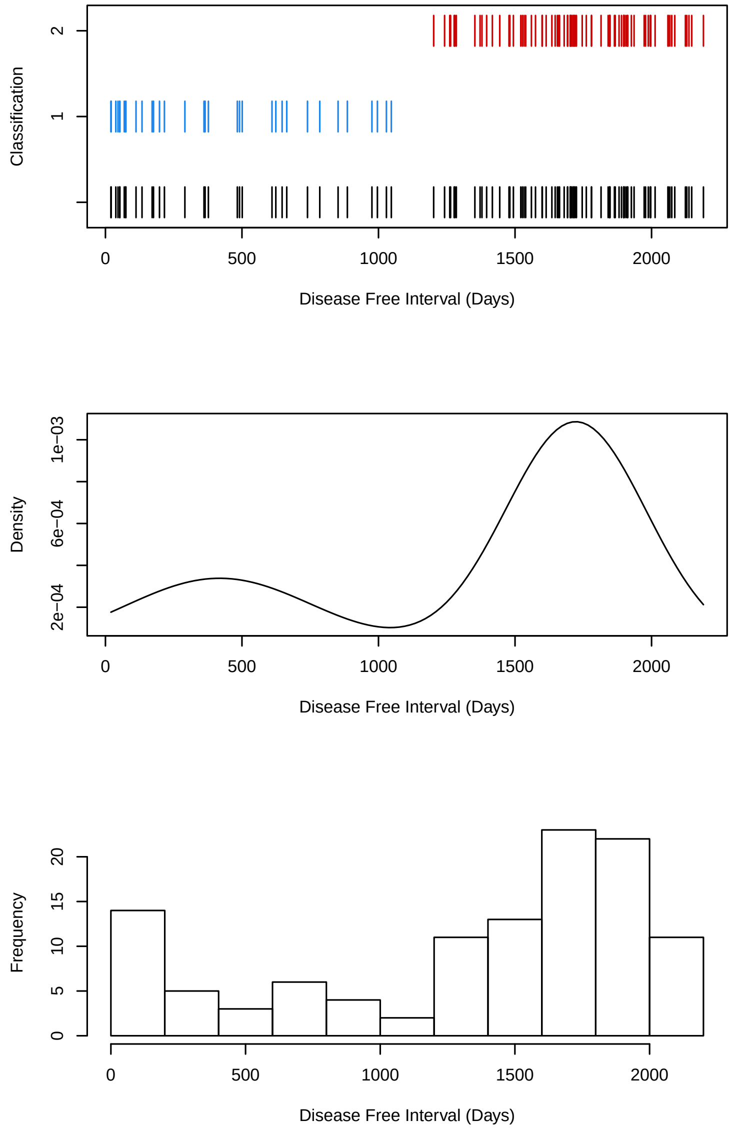

Figure S10: A. Classification of UCEC patients in short DFI (blue) and long DFI (red). B. 2-component Gaussian mixture density corresponding to the above classification. C. Histogram of DFI for patients.

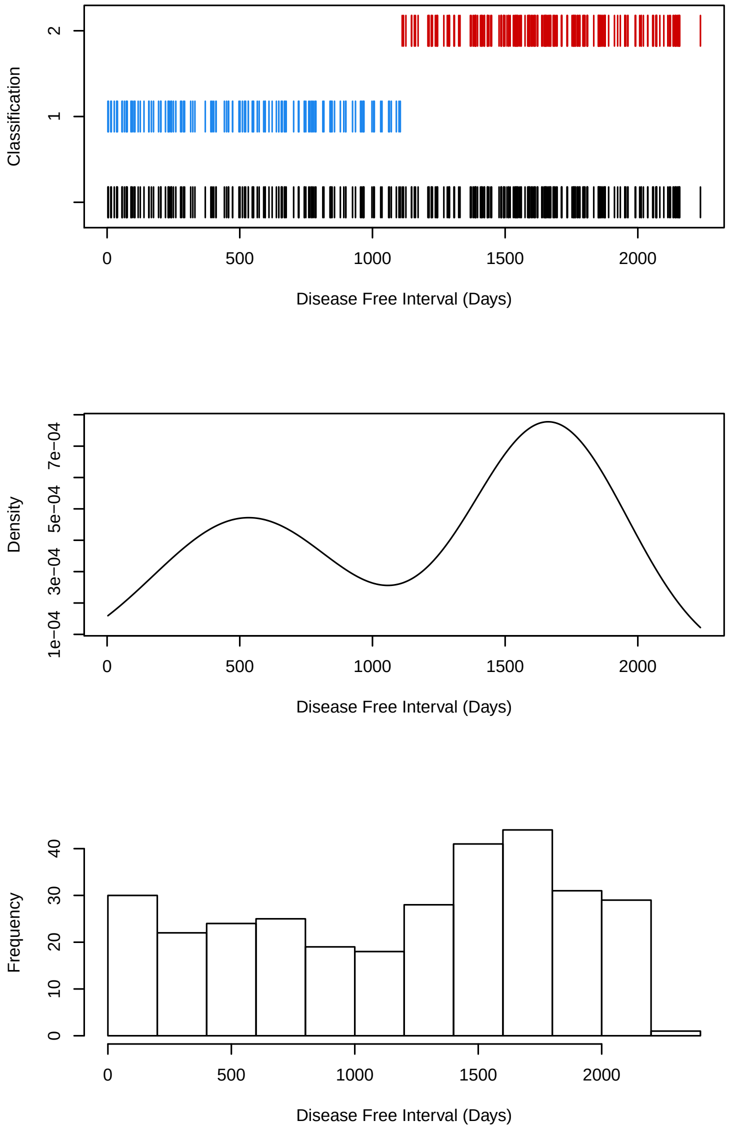

Figure S11: A. Classification of LIHC patients in short DFI (blue) and long DFI (red). B. 2-component Gaussian mixture density corresponding to the above classification. C. Histogram of DFI for patients.

References

[1] Michael Ashburner, Catherine A Ball, Judith A Blake, David Botstein, Heather Butler, J Michael Cherry, Allan P Davis, Kara Dolinski, Selina S Dwight, Janan T Eppig, et al. Gene ontology: tool for the unification of biology. *Nature genetics*, 25(1):25–29, 2000.

[2] Domokos Bartis, Veronika Csongei, Alexander Weich, Edit Kiss, Szilvia Barko, Tamas Kovacs, Monika Avdicevic, Vijay K D'Souza, Judit Rapp, Krisztian Kvell, et al. Down-regulation of canonical and up-regulation of non-canonical wnt signalling in the carcinogenic process of squamous cell lung carcinoma. *PloS one*, 8(3), 2013.

[3] Harald Binder, Christine Porzelius, and Martin Schumacher. An overview of techniques for linking high-dimensional molecular data to time-to-event endpoints by risk prediction models. *Biometrical Journal*, 53(2):170–189, 2011.

[4] Dawn T Bravo, Yi-Lin Yang, Kristopher Kuchenbecker, Ming-Szu Hung, Zhidong Xu, David M Jablons, and Liang You. Frizzled-8 receptor is activated by the wnt-2 ligand in non-small cell lung cancer. *BMC cancer*, 13(1):316, 2013.

[5] Ricardo J. G. B. Campello, Davoud Moulavi, and Joerg Sander. Density-Based Clustering Based on Hierarchical Density Estimates. In Jian Pei, Vincent S. Tseng, Longbing Cao, Hiroshi Motoda, and Guandong Xu, editors, *Advances in Knowledge Discovery and Data Mining*, Lecture Notes in Computer Science, pages 160–172, Berlin, Heidelberg, 2013. Springer.

[6] Hung-Chia Chen, Ralph L Kodell, Kuang Fu Cheng, and James J Chen. Assessment of performance of survival prediction models for cancer prognosis. *BMC medical research methodology*, 12(1):102, 2012.

[7] Antonio Colaprico, Tiago C Silva, Catharina Olsen, Luciano Garofano, Claudia Cava, Davide Garolini, Thais S Sabedot, Tathiane M Malta, Stefano M Pagnotta, Isabella Castiglioni, et al. Tcgabiolinks: an r/bioconductor package for integrative analysis of tcga data. *Nucleic acids research*, 44(8):e71–e71, 2016.

[8] Gene Ontology Consortium. The gene ontology resource: 20 years and still going strong. *Nucleic acids research*, 47(D1):D330–D338, 2019.

[9] Geoffrey M Cooper and RE Hausman. A molecular approach. *The Cell. 2nd ed. Sunderland, MA: Sinauer Associates*, 2000.

[10] Michelino De Laurentiis, Sabino De Placido, Angelo R Bianco, Gary M Clark, and Peter M Ravdin. A prognostic model that makes quantitative estimates of probability of relapse for breast cancer patients. *Clinical Cancer Research*, 5(12):4133–4139, 1999.

[11] Amit Dutt, Helga B Salvesen, Tzu-Hsiu Chen, Alex H Ramos, Robert C Onofrio, Charlie Hatton, Richard Nicoletti, Wendy Winckler, Rupinder Grewal, Megan Hanna, et al. Drug-sensitive fgfr2 mutations in endometrial carcinoma. *Proceedings of the National Academy of Sciences*, 105(25):8713–8717, 2008.

[12] Martin Ester, Hans-Peter Kriegel, Jörg Sander, Xiaowei Xu, et al. A density-based algorithm for discovering clusters in large spatial databases with noise. In *Kdd*, volume 96, pages 226–231, 1996.

[13] Dmitriy Fradkin and Ilya Muchnik. Support vector machines for classification. *DIMACS series in discrete mathematics and theoretical computer science*, 70:13–20, 2006.

[14] Florian R Greten and Sergei I Grivennikov. Inflammation and cancer: Triggers, mechanisms, and consequences. *Immunity*, 51(1):27–41, 2019.

[15] Yucheng Guo, Qing Nie, Adam L MacLean, Yanda Li, Jinzhi Lei, and Shao Li. Multiscale Modeling of Inflammation-Induced Tumorigenesis Reveals Competing Oncogenic and Oncoprotective Roles for Inflammation. *Cancer Research*, 77(22):6429–6441, November 2017.

[16] Zhibin Hu, Xi Chen, Yang Zhao, Tian Tian, Guangfu Jin, Yongqian Shu, Yijiang Chen, Lin Xu, Ke Zen, Chenyu Zhang, et al. Serum microrna signatures identified in a genome-wide serum microrna expression profiling predict survival of non-small-cell lung cancer. *J Clin Oncol*, 28(10):1721–1726, 2010.

[17] John P. Klein, Hans C. Van Houwelingen, Joseph G. Ibrahim, and Thomas H. Scheike. *Handbook of survival analysis.* CRC Press, 2016.

[18] James E Korkola, Jane Houldsworth, Darren R Feldman, Adam B Olshen, Li-Xuan Qin, Sujata Patil, Victor E Reuter, George J Bosl, and RSK Chaganti. Identification and validation of a gene expression signature that predicts outcome in adult men with germ cell tumors. *Journal of Clinical Oncology*, 27(31):5240, 2009.

[19] N Kramer, J Schmöllerl, C Unger, H Nivarthi, A Rudisch, D Unterleuthner, M Scherzer, A Riedl, M Artaker, I Crncec, et al. Autocrine wnt2 signaling in fibroblasts promotes colorectal cancer progression. *Oncogene*, 36(39):5460–5472, 2017.

[20] Kwan-Moon Leung, Robert M Elashoff, and Abdelmonem A Afifi. Censoring issues in survival analysis. *Annual review of public health*, 18(1):83–104, 1997.

[21] Arthur Liberzon, Chet Birger, Helga Thorvaldsdóttir, Mahmoud Ghandi, Jill P Mesirov, and Pablo Tamayo. The molecular signatures database hallmark gene set collection. *Cell systems*, 1(6):417–425, 2015.

[22] Jo-Han Lim, Kyeong-Eun Lee, Kyu-S Hahn, and Kun-Woo Park. Analyzing survival data as binary outcomes with logistic regression. *Communications for Statistical Applications and Methods*, 17(1):117–126, 2010.

[23] Jianfang Liu, Tara Lichtenberg, Katherine A Hoadley, Laila M Poisson, Alexander J Lazar, Andrew D Cherniack, Albert J Kovatich, Christopher C Benz, Douglas A Levine, Adrian V Lee, et al. An integrated tcga pan-cancer clinical data resource to drive high-quality survival outcome analytics. *Cell*, 173(2):400–416, 2018.

[24] William W Lockwood, Sahiba K Chandel, Greg L Stewart, Hediye Erdjument-Bromage, and Levi J Beverly. The novel ubiquitin ligase complex, scffbxw4, interacts with the cop9 signalosome in an f-box dependent manner, is mutated, lost and under-expressed in human cancers. *PloS one*, 8(5), 2013.

[25] Leland McInnes, John Healy, and James Melville. Umap: Uniform manifold approximation and projection for dimension reduction. *arXiv preprint arXiv:1802.03426*, 2018.

[26] Amanda J Mikels and Roel Nusse. Purified wnt5a protein activates or inhibits β-catenin–tcf signaling depending on receptor context. *PLoS biology*, 4(4), 2006.

[27] Virginia Murillo-Garzón, Irantzu Gorroño-Etxebarria, Malin Åkerfelt, Mikael Christer Puustinen, Lea Sistonen, Matthias Nees, James Carton, Jonathan Waxman, and Robert M Kypta. Frizzled-8 integrates wnt-11 and transforming growth factor-β signaling in prostate cancer. *Nature communications*, 9(1):1–16, 2018.

[28] Manfred Opper and Ole Winther. Gaussian processes for classification: Mean-field algorithms. *Neural computation*, 12(11):2655–2684, 2000.

[29] Lisette G. de Pillis, Ami E. Radunskaya, and Charles L. Wiseman. A Validated Mathematical Model of Cell-Mediated Immune Response to Tumor Growth. *Cancer Research*, 65(17):7950–7958, September 2005.

[30] Carl Edward Rasmussen and Christopher KI Williams. Gaussian processes for machine learning, vol 14, no 2, 2006.

[31] David Ricol, David Cappellen, Ahmed El Marjou, Sixtina Gil-Diez-de Medina, Jeanne-Marie Girault, Teruhiko Yoshida, Gilles Ferry, Gordon Tucker, Marie-France Poupon, Dominique Chopin, et al. Tumour suppressive properties of fibroblast growth factor receptor 2-iiib in human bladder cancer. *Oncogene*, 18(51):7234–7243, 1999.

[32] Mark Robertson-Tessi, Ardith El-Kareh, and Alain Goriely. A mathematical model of tumor–immune interactions. *Journal of Theoretical Biology*, 294:56–73, February 2012.

[33] Adriana Sánchez-Danés, Jean-Christophe Larsimont, Mélanie Liagre, Eva Muñoz-Couselo, Gaëlle Lapouge, Audrey Brisebarre, Christine Dubois, Mariano Suppa, Vijayakumar Sukumaran, Véronique Del Marmol, et al. A slow-cycling lgr5 tumour population mediates basal cell carcinoma relapse after therapy. *Nature*, 562(7727):434, 2018.

[34] Jarno Vanhatalo, Jaakko Riihimäki, Jouni Hartikainen, Pasi Jylänki, Ville Tolvanen, and Aki Vehtari. Gpstuff: Bayesian modeling with gaussian processes. *Journal of Machine Learning Research*, 14(Apr):1175–1179, 2013.

[35] Aki Vehtari, Andrew Gelman, and Jonah Gabry. Practical bayesian model evaluation using leave-one-out cross-validation and waic. *Statistics and computing*, 27(5):1413–1432, 2017.

[36] Hanbo Wang, Leilei Niu, Shaobo Jiang, Jing Zhai, Ping Wang, Feng Kong, and Xunbo Jin. Comprehensive analysis of aberrantly expressed profiles of lncrnas and mirnas with associated cerna network in muscle-invasive bladder cancer. *Oncotarget*, 7(52):86174, 2016.

[37] Daniela M Witten and Robert Tibshirani. Survival analysis with high-dimensional covariates. *Statistical methods in medical research*, 19(1):29–51, 2010.
